# The RPN12a proteasome subunit is essential for the multiple hormonal homeostasis controlling the progression of leaf senescence

**DOI:** 10.1038/s42003-022-03998-2

**Published:** 2022-09-30

**Authors:** Clément Boussardon, Pushan Bag, Marta Juvany, Jan Šimura, Karin Ljung, Stefan Jansson, Olivier Keech

**Affiliations:** 1 Department of Plant Physiology, Umeå Plant Science Centre, Umeå University, SE-901 87 Umeå, Sweden; 2 Department of Forest Genetics and Plant Physiology, Umeå Plant Science Centre, Swedish University of Agricultural Sciences, SE-901 83 Umeå, Sweden

**Keywords:** Leaf development, Senescence

## Abstract

The 26S proteasome is a conserved multi-subunit machinery in eukaryotes. It selectively degrades ubiquitinated proteins, which in turn provides an efficient molecular mechanism to regulate numerous cellular functions and developmental processes. Here, we studied a new loss-of-function allele of RPN12a, a plant ortholog of the yeast and human structural component of the 19S proteasome RPN12. Combining a set of biochemical and molecular approaches, we confirmed that a *rpn12a* knock-out had exacerbated 20S and impaired 26S activities. The altered proteasomal activity led to a pleiotropic phenotype affecting both the vegetative growth and reproductive phase of the plant, including a striking repression of leaf senescence associate cell-death. Further investigation demonstrated that RPN12a is involved in the regulation of several conjugates associated with the auxin, cytokinin, ethylene and jasmonic acid homeostasis. Such enhanced aptitude of plant cells for survival in *rpn12a* contrasts with reports on animals, where 26S proteasome mutants generally show an accelerated cell death phenotype.

## Abstract (web summary)

A loss-of-function mutation in the RPN12a proteasome subunit in *Arabidopsis thaliana* shows that this protein is essential for hormonal homeostasis, thereby modulating plant’s development and the progression of leaf senescence.

## Introduction

Past research has shown that the molecular mechanisms controlling the induction and progression of cell death diverge significantly between animals and plants. For instance, while the cell death-dependent molecular mechanisms associated with senescence applies to both kingdoms, plants possess a particular type of cell death mechanism, defined as a vacuolar-type cell death^1^, which allows for the specific recovery and export of nutrients from the senescing organ towards the rest of the plant. Leaf senescence is undoubtedly the most well-studied developmental process when it comes to senescence in plants, as it contributes actively to plant fitness and seed quality. With the aim to better understand the molecular mechanisms controlling the induction and progression of leaf senescence, we performed a genetic screen to isolate functional stay-green mutants in *Arabidopsis thaliana*^2^ (Arabidopsis). One of these EMS (ethyl methanesulfonate) mutants, named *FSG-236*, was supposedly associated with a dysfunctional proteasome.

The 26S proteasome is a very well conserved multi-subunit machinery in eukaryotes. It selectively degrades tagged proteins, which in turn provides an efficient molecular mechanism to regulate numerous cellular functions and developmental processes. The 26S proteasome consists of a 20S core particle (CP) and a 19S regulatory particle (RP) located at one or both extremities of the 20S CP; in the latter case only one of the two 19S particles is functional^3,4^. Proteins subjected to a tight regulation are first ubiquitinated, and often referred to as ubiquitin-protein conjugates (Ubn–protein conjugates), by a wide range of specific E3 ligases^5^. Subsequently, the Ubn–protein conjugates are recognized by the 19S, which encompasses two sub-particles named base and lid. The base is composed of six regulatory particle AAA ATPase subunits (RPTs) and three regulatory particles non-ATPase (RPN1, RPN2, and RPN10), the latter being necessary for the identification of ubiquitinated targets. The lid is composed of eight RPN subunits and insures the substrate recognition and de-ubiquitination function^6,7^. Following de-ubiquitination, proteins are unfolded and translocated to the 2S CP, a hepta-subunits inner β-ring that operates the proteolytic activity via β1, β2, and β5^8–10^.

Here, we studied a loss of function allele of RPN12a, a plant ortholog of the yeast and human structural component of the 19S proteasome RPN12. In line with previous studies made on other RP mutants^11,12^, we showed that a *rpn12a* knock-out had exacerbated 20S and impaired 26S activities, which led to a phenotypical pleiotropy including a striking repression of leaf senescence. Further investigation demonstrated that RPN12a is essential for the maintenance of homeostasis between multiple hormone conjugates.

## Results

### A premature stop codon in the *RPN12a* coding sequence leads to a functional stay-green phenotype

**Fig. 1:**
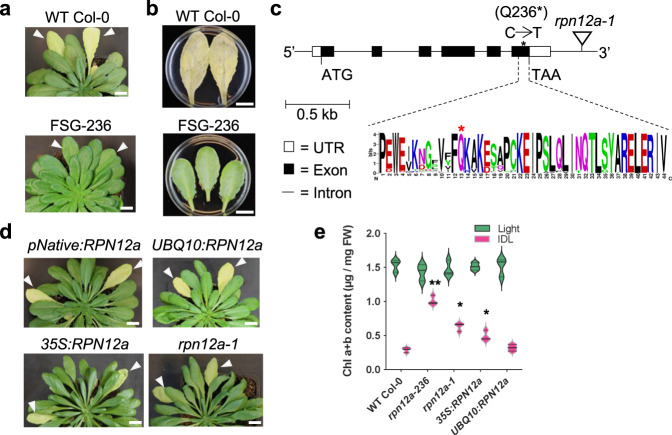
Molecular and phenotypic characterization of the EMS mutant Arabidopsis mutants subjected to dark-induced senescence. **a** Senescence and stay-green phenotype of WT Col-0 and EMS mutant leaves individually darkened (IDL) for 7 days (white arrows), respectively, and **b** of detached leaves from 6-week-old plants, grown in SD, placed in darkness for 7 days in 3 mM MES (pH 5.7). **c** Localization of the EMS mutation in the *RPN12a* gene causing the stay-green phenotype due to a premature stop codon (Q236*) in the C-terminal end of the RPN12a protein. White boxes: UTR, dark boxes: exons, and lines: introns. Weblogo was used to visualize the sequence conservation in the 187 closest proteins aligning with the last 44 amino acids in the C-terminal region of RPN12a. The height of each letter (score on *y*-axis) indicates the relative frequency of an amino acid at a given position, whilst the overall height of the stack indicates sequence conservation at that position. Red asterisk (*): glutamine in position 236 which is turned into a stop codon. **d** Phenotype of individually darkened leaves (7 days) in two complemented lines (driven by a UBQ10 or 35S promotor), and a *rpn12a-1* T-DNA mutant. **e** Chlorophyll content of the *rpn12a* mutants and complemented lines (from 6-week-old plants, grown in SD). The violin plots show a medium smoothing of the distribution of the data with the median (plain line) and two quartiles (dotted line); a Student’s *t*-test (two sided; *n* = 3 biologically independent replicates) showed statistically significant differences from WT Col-0 for similar treatment at *p < 0.05, **p < 0.001. Scale bars = 1 cm.

With the aim to identify new key members regulating the induction and progression of cell death during senescence in leaves, we carried out a screen for a delayed dark-induced senescence phenotype (DIS)^2^ in EMS-mutagenized Arabidopsis Columbia (Col-0) plants in the M2 generation. This was assessed using the individually darkened leaves (IDL) experiment^13^. In short, while the plant is exposed to light, two leaves were placed in darkness, which triggers a synchronized induction of senescence. After 7 days, IDL of the recessive mutant, named *FSG-236*, showed a delayed senescence while IDLs of WT turned yellow (Fig. 1a). A similar phenotype was observed with detached leaves placed on a floating medium and kept in darkness for 7 days, which indicates that the functional stay-green (FSG) phenotype is coupled to the endogenous metabolism of the cells from the leaf itself and does not result from a signaling event between different parts of the plant (Fig. 1b). The plants were self-fertilized to the M4 generation, when the delay of senescence trait was fixed, and backcrossed twice to clean the genetic background.

To identify the mutation responsible for the FSG phenotype, sequencing on a bulk of eight backcrossed plants was carried out and all SNPs were identified. We narrowed down our search for causal mutations by identifying exon-coded, non-synonymous, and homozygous mutations that turns G-to-A or C-to-T. Seven different genes on chromosome 1, each containing a single non-synonymous point mutation, were identified (Supplementary Data 1). One of these, a C-to-A mutation, turned a glutamine (Q_236_) to an early stop codon in the sequence of the RPN12a 26S proteasome regulatory particle subunit, which potentially led to a truncated protein lacking 32 amino acids in its C-terminal region (Fig. 1c). RPN12a is well conserved between species. The RPN12 of fission yeast (*Schizosaccharomyces pombe*), an ortholog of AtRPN12a, is composed of N-terminal tetratricopeptide repeats and a winged helix (WH) domain in the C-terminal. The RPN12 WH domain of *S. pombe* is aligned with residues D_185_ to V_234_ of Arabidopsis RPN12a protein, hence, potentially still present in the mutant version of RPN12a^14^. To get a better understanding of the conservation of the RPN12a C-terminal end in plants, 187 protein sequences from different plants were identified using blastP using the last 44 amino acids of RPN12a sequence as probe. A sequence logo representation of the conservation of the C-terminal part of RPN12a across this dataset is shown in Fig. 1c. The 32 amino acids sequence in the C-terminal end is remarkably well conserved within species suggesting an important role for the deleted RPN12a domain in the mutant.

To confirm that the FSG phenotype observed in the mutants was a consequence of the premature stop codon in *RPN12a*, the mutant was complemented with the *RPN12a* open reading frame (ORF) under the control of constitutive promoters, 35S and UBQ10, or with a 2963 bp genomic fragment comprising a region of 861 bp before ATG, the *RPN12a* ORF (1662 bp) and 440 bp downstream. After transformation, IDL experiments were conducted on T2 complemented lines for each construct. Restoration of a WT-like progression of senescence was observed for complemented lines containing the *UBQ10:RPN12a* and the 2963 bp genomic fragment transgenes (Fig. 1d). Only a partial rescue was observed for the *35S:RPN12a* construct, and the phenotype was similar to the *rpn12a-1* mutant^15^(Fig. 1d). The *rpn12a-1* mutant contains an ARP-NPTII T-DNA insertion downstream of *RPN12a*, which impedes its expression but does not lead to a true knockout. This comparison thus suggests that *35S:RPN12a* construct had been partially silenced. We also quantified the chlorophyll content in all these lines in both IDL and light controls (Fig. 1e). As expected, the EMS mutant, hereafter named *rpn12a-236*, retained more chlorophyll than the *rpn12a-1* mutant and *35S:RPN12a* complemented line. The WT and *UBQ10:RPN12a* complemented line had a strong reduction in their chlorophyll content upon the darkening treatment confirming the senescence phenotype observed in IDL after 7 days (Fig. 1e). Altogether, this demonstrated that the premature stop codon in *RPN12a* caused the changed induction and progression of senescence-associated cell death in response to the stress “prolonged darkness”.

### The *rpn12a-236* mutant displays a pleiotropic developmental phenotype

**Fig. 2:**
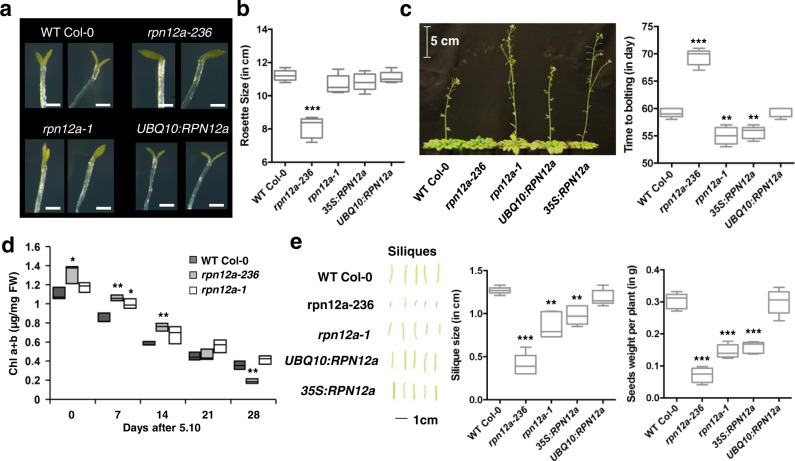
Phenotypic characterization of *rpn12a-*236 during growth and development and comparison with other lines. **a** Skotomorphogenesis of seedlings after 10 days in darkness. *rpn12a-236* exhibits a reduced cotyledon opening and a marked apical hook. Scale bars = 0.1 cm. **b** Rosette diameter, and **c** Time to bolting measured at 5.10 developmental stage^18^ for plants grown under short-day conditions (*n* = 5 biologically independent replicates per genotype) **d** Chlorophyll content in leaves (rosette 7 to 12) undergoing developmental senescence (*n* = 3 biologically independent replicates per genotype; box plots are min max and the middle line is the median). **e** Silique growth and seed production for all studied genotypes. (*n* = 100 (i.e., 5 biologically independent replicates with 20 siliques each). For box and whiskers plots in **b**, **c**, and **e**, the interquartile range of the box is from the 25th to 75th percentiles, and the middle line is the median; whiskers represent min and max values; a Student’s *t*-test (two sided) showed statistically significant differences at *p < 0.05, **p < 0.001, ***p < 0.0001 when compared to WT.

We tested whether additional developmental phenotypes were observed in the *rpn12a-236* mutant and how it compared with the previously characterized *rpn12a-1* T-DNA mutant^15^. While both the *rpn12a-236* and *rpn12a-1* mutants showed a delay in skotomorphogenesis compared to the WT and *UBQ10:RPN12a* complemented line when seedlings were grown in the dark, a more severe delay in cotyledon and apical hook opening was observed in *rpn12a-236* than *rpn12a-1* (Fig. 2a). Under short-day (SD) growth conditions, a strong reduction of rosette size and a delay in the transition from vegetative to reproductive growth was observed in *rpn12a-236* as compared with WT and *UBQ10:RPN12a*, that needed on average 59.4 and 60.2 days before bolting, respectively, compared to ca. 70 days in *rpn12a-236* (Fig. 2b, c). Unexpectedly, both *rpn12a-1* and the *35S:RPN12a* complemented line bolted significantly earlier. Since vegetative growth was significantly delayed in the *rpn12a-236* mutant, developmental senescence also logically appeared delayed when assessed on a time basis. However, when estimated after the 5.10 developmental stage^16^, progression of developmental senescence appeared much quicker in *rpn12a-236* than in the WT and *rpn12a-1* plants, suggesting a dysfunctional regulation of this developmental process in *rpn12a-236* (Fig. 2d and Fig. S1). In line with this, siliques of *rpn12a-236* were underdeveloped (3-fold shorter than WT), albeit fertile, and less productive than in the other genotypes tested: with about 2-times lower seed yield than in *rpn12a-1* and *35S:RPN12a*, and more than 4-times lower than *UBQ10:RPN12a* and WT (Fig. 2e). Taken together, these results show that *rpn12a-236* has a pleiotropic phenotype, including a retarded growth and a much lower seed production than the WT and the leaky allele *rpn12a-1*.

### The proteasome activity is altered in *rpn12a-236*

**Fig. 3:**
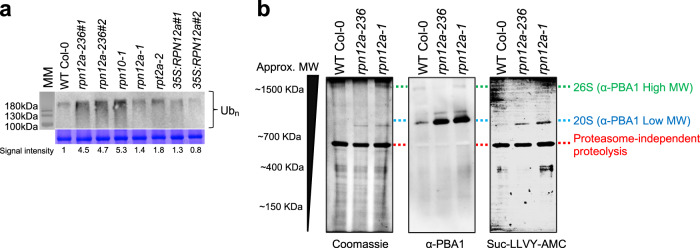
Proteasome assembly and activity. **a** Levels of Ubn–protein conjugates in WT Col-0*, rpn12a-1*, *rpt2a-2, rpn10-1*, *rpn12a-236,* and *35S:RPN12a* leaves. Twenty micrograms of total protein (control = Coomassie blue staining with a focus on Rubisco large subunit) were subjected to SDS-PAGE and immunoblot analysis with anti-ubiquitin antibodies. The signal intensity of ubiquitin-protein conjugates was estimated, and fold-change as compared to WT Col-0 was reported below the immunoblot for each genotype. **b** Proteasome chymotrypsin-like activity. Native-PAGE followed by immunoblot analyses of the PBA1 subunit (middle) and activity test (right) in 10 µg of total protein extracts from 10-day-old seedlings. For the 20S proteasome activity, native-PAGE membrane was incubated with a Suc-LLVY-AMC fluorophore solution and revealed at 365 nm. Loading control was checked by Coomassie staining (left) (experiments were run in triplicates from independent extracts).

To test the extent to which this deletion causes stability issues in the truncated protein of *rpn12a-236*, we performed an immunoblot assay with the RPN12a antibody on *rpn12a-236* and several proteasome mutants: *rpn12a-1*, *rpt2a-2*, and *rpn10-1*. RPN12a was translated in all mutant lines including *rpn12a-236*, although the signal was hardly detectable in *rpn12a-1*, as previously described^15^ (Fig. S2). This indicates that the C-terminal deletion does not affect protein stability, and thus the *rpn12a-236* mutant version of RPN12a would potentially still be incorporated into the 26S proteasome complex. Therefore, to assess the ubiquitin-dependent activity of the 26S proteasome, we compared the Ubn–protein profiles of WT, *rpn12a-236*, *rpn12a-1*, *rpt2a-2*, and *rpn10-1*; the latter two being subunits of the 19S proteasome but where only *rpn10-1* is known to accumulate Ubn-proteins^11,17^. Both *rpn10-1* and the *rpn12a-236* mutants had levels of Ubn-proteins ca. 4 to 5-fold higher than all the other genotypes (Fig. 3a), suggesting a strong impairment of the 26S proteasome functionality in *rpn12a-236* (and in *rpn10-1*) as compared to the *rpn12a-1*.

Consequently, we examined the 26S proteasome assembly of *rpn12a-236* on a one-dimension native-PAGE followed by an immunoblot of the 20S CP subunit PBA1. The PBA1 signal corresponding to the 20S CP was 7-fold stronger in both *RPN12a* mutants than in WT (Fig. 3b), showing that the 20S proteasome accumulated in the *RPN12a* 19S RP mutants. Furthermore, while the 26S proteasome signal was visible and of similar intensity in *rpn12a-1* and WT, it could barely be detected in the *rpn12a-236* mutant (Fig. 3b). We therefore concluded that the accumulation of the Ubn–protein conjugates in *rpn12a-236* is correlated to a change in the 26S/20S proteasomes ratio. This was confirmed by running immunoblotting on a second-dimension gel. Immunoblots against PBA1 and RPN12a were used to determine the localization of the 20S CP and the 19S, respectively (Fig. S3). Finally, the proteasome chymotrypsin-like activity was checked using Suc-LLVY-AMC, a fluorogenic substrate that is cleaved by the β5 subunit of the 20S proteasome. As seen on membrane and in vitro, the chymotrypsin-like activity of *rpn12a-236* and *rpn12a-1* was higher than in the WT, demonstrating an enhanced 20S proteasome activity in the two *rpn12a* mutants (Fig. 3b and Fig. S4). Of note, on membrane, a strong and stable proteasome independent (i.e., which does not react with PBA1 and RPN12a antibodies) AMC signal corresponding to a ca. 600 kDa complex was detected (Fig. 3b). Second-dimension Ponceau staining showed that the signal colocalized with a 65 kDa protein (Fig. S3), suggesting that this protein could form a multimer containing a chymotrypsin-like protease activity.

It has previously been observed that oxidized proteins are preferential targets of the 20S particle, and that 19S particle mutants showed an enhanced non-ubiquitin-dependent protein degradation as well as an increased oxidative stress tolerance^11^. Furthermore, it is known that oxidized proteins accumulate during the progression of leaf senescence^18,19^. Since *rpn12a-236* had more 20S, we hypothesized that the FSG phenotype observed in *rpn12a-236* could be linked, at least partially, to an enhanced tolerance to ROS-induced oxidative stress. To this end, we estimated the amount of hydrogen peroxide (H_2_O_2_) and superoxide (O_2_^−^) using Diaminobenzidine (DAB) and Nitro Blue Tetrazolium (NBT), respectively, in IDLs of WT and *rpn12a-236*. After 3 days and 6 days of darkening treatment, leaves of *rpn12a-236* showed only scarce DAB brown color while in WT, the DAB coloration first decreased after 3 days but accumulated massively after 6 days in darkness (Fig. S5a). Conversely, the NBT signal decreased in the WT after 3 days and 6 days of darkness when compared to the light control, while it remained stable in *rpn12a-236* across the time-course of the treatment (Fig. S5b). We thus concluded that the darkening treatment led to a decrease in H_2_O_2_ accumulation in *rpn12a-236* mutant leaves, contrasting with the increased DAB signal observed in WT during the progression of senescence

Finally, as protein carbonylation is a marker of the damaging effect of ROS^19^, we determined the level of carbonylated proteins in the WT and *rpn12a-236* proteasome mutant leaves using 2,4-dinitrophenylhydrazine (DNPH) derivatization. The *rpn12a-236* mutant displayed a stable amount of anti-DNP signal over time, suggesting that the carbonylation of proteins was not induced in IDLs, whereas a 2.6-fold accumulation was detected in WT IDLs after 6 days (Fig. S5c). Altogether, we concluded that the *rpn12a-236* mutant had a compromised 26S proteasome activity, likely with lower processing of the Ubn–protein conjugates and enhanced 20S activity, which led to a reduced cell death-induced oxidative stress.

### RPN12a undergoes a profound transcriptional shift both in illuminated and individually darkened leaves

**Fig. 4:**
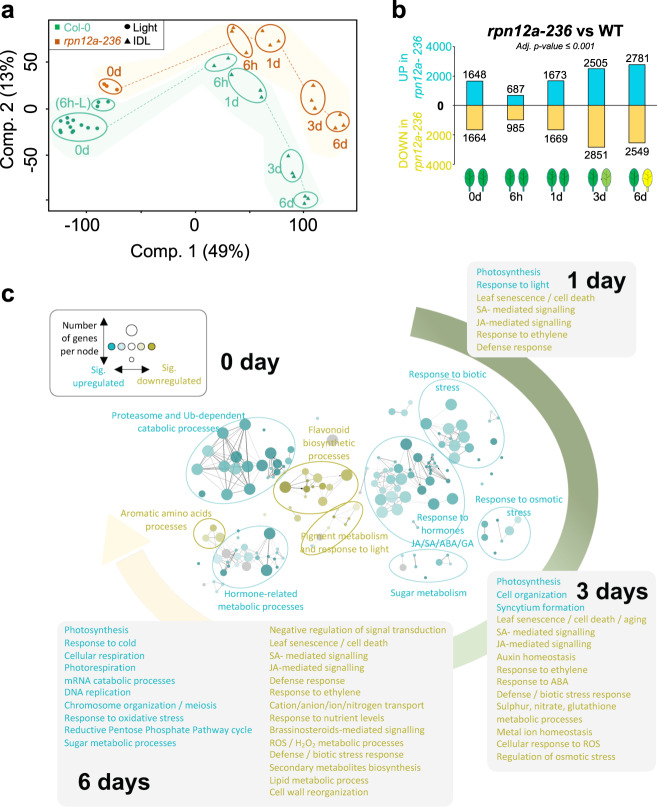
Transcriptomic analysis of WT Col-0 and *rpn12a-236* in response to IDL treatment. **a** Resemblances and variations within the RNA-seq samples were observed by PCA; first component (49%) and second component (13%). **b** Differential expression analysis (*P* ≤ 0.001) between WT and mutant across the IDL time-course. Number of genes up- and downregulated between the two genotypes at a given time point are shown above and under the histograms, respectively. **c** Biological network of enriched GO terms (Biological Process) from significantly (*p* < 0.001) differentially expressed genes in *rpn12a-236* versus WT. Enriched GO terms upregulated in *rpn12a-236* (palette of blues) or downregulated (palette of yellows) were clustered according to their functional categories. The size of a node is proportional to the number of genes contributing to this node, while the intensity of the color is proportional to the significance of it (the minimum for significance being *p* < 0.05); gray nodes = overrepresented in the gene subset but not enriched significantly; the threshold of significance was set at 60%. For day 1, 3, and 6, the subset of genes differentially regulated at day 0 was subtracted to highlight the functional categories associated with cell death and minimize the constitutive contribution of a non-functional proteasome (see Methods).

To gain insight into the molecular mechanisms conferring this extended longevity to *rpn12a-236* in response to darkness, we performed RNA-seq analysis on WT and mutant leaves sampled after 0 h (light control), 6 h, 1 day, 3 days, and 6 days of IDL treatment. We plotted the first two components of a Principal Component Analysis (PCA) from normalized RNA-seq-data, and observed that the first component, accounting for 49% of the variance in the experiment, reflected the response to darkness and the treatment time-course (Fig. 4a). Components 2 and 3, accounting for 13% and 10% of the variance, respectively, separated for time and genetic background (Fig. 4a and Fig. S6). These plots revealed that for both genotypes, IDLs had a comparable trend in their response to darkness, yet diverged by the amplitude of this response. More than 3000 genes were differentially expressed already at 0 day, i.e., before the IDL treatment (*p* ≤ 0.001) (Fig. 4b). This observation was not surprising considering the *rpn12a-236* pleiotropic developmental phenotype. Curiously, the number of differentially expressed genes (DEGs) dropped to around 1500 after a 6 h treatment, suggesting an influence of the circadian rhythm on to the proteasome-dependent regulation of cell metabolism. After 6 days, more than 5000 genes were differentially regulated in IDL of *rpn12a-236* as compared to IDL of WT (Fig. 4b).

To better understand the reasons for this high number of DEGs, we established a biological network based on gene ontology (GO) terms from significantly DEGs (*p* ≤ 0.001) using the ClueGO plugin^20^ for Cytoscape^21^. The network provides an overview of the GO terms for Biological Process up- and downregulated in *rpn12a-236* as compared to WT at *T* = 0 day, and a selection of the significantly enriched GO terms for 1 day, 3 days, and 6 days. GO terms were indicated in the boxes (Fig. 4c; see Methods). This approach clearly identified a series of biological functions differentially regulated between the two genotypes. For example, at 0 day, a strong enrichment in GO terms associated with proteasome and Ub-dependent catabolic processes, hormone- and sugar-related metabolic processes, and response to stress were seen in *rpn12a-236*, whereas aromatic amino acids, pigment metabolism and flavonoid biosynthetic processes GO terms were positively correlated with the WT (Fig. 4c). Through the progression of DIS, the enriched GO terms downregulated in *rpn12a-236* (i.e., positively correlated with WT) delineated a set of biological processes associated with leaf senescence, including numerous catabolic processes, a response to oxidative stress, and pathways related to hormone homeostasis and signaling; notably ethylene (ET), abscisic acid (ABA), jasmonic acid (JA), salicylic acid (SA), and auxin (IAA). In contrast, the vast majority of the enriched GO terms in the mutant were linked to photosynthesis, respiration, and a response to oxidative stress. To summarize, this network analysis of GO terms clearly indicated that the FSG phenotype observed in *rpn12a-236* was linked to a modulation of transcripts associated with proteasomal activity, the maintenance of the primary metabolism, the impaired progression of senescence/cell death-associated molecular mechanisms (e.g., catabolic processes, oxidative stress, transport), and hormonal biosynthesis and signaling processes.

### Mutation in RPN12a 19S particle subunits lead to the modulation of senescence-responsive genes linked to hormones signaling

The results of our GO analysis prompted us to perform detailed transcript profiling of genes coding for the proteasome subunits; of senescence-responsive genes; and of genes involved in hormones biosynthesis, conjugation, and signaling.

**Fig. 5:**
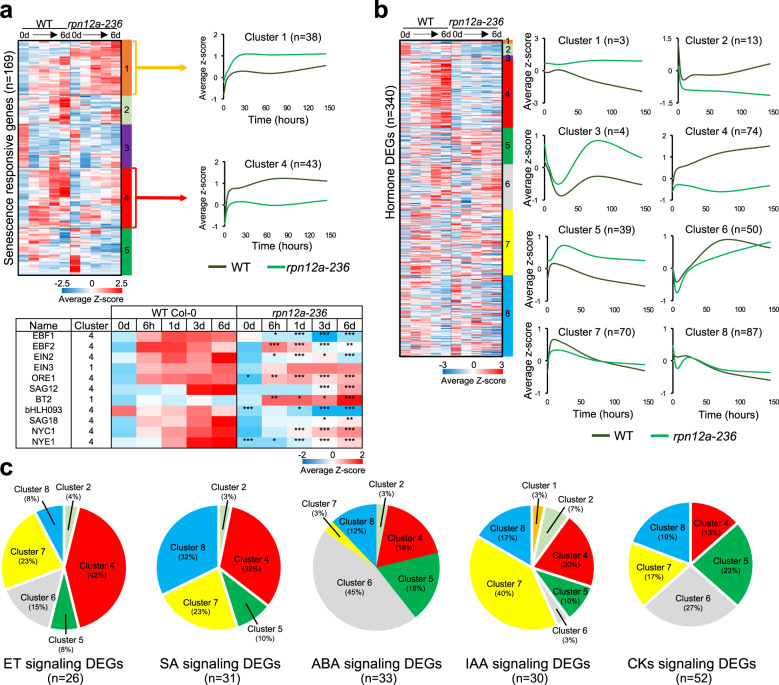
Senescence- and hormones metabolism-associated genes expression analysis. **a** Analysis of senescence-associated genes. Top left: the heatmap was divided in 5 clusters defined by hierarchical clustering using MeV (Pearson correlation). Top right: the graphs depict the overall abundance pattern of transcripts for WT and *rpn12a-236* in the two selected clusters. Average *Z*-score was plotted overtime for each cluster. Bottom: expression profile of key genes involved in senescence and found in clusters 1 and 4. A Student’s *t*-test (two sided) showed statistically significant differences at *p < 0.01, **p < 0.001, ***p < 0.0001. **b** Hierarchical clustering of DEGs associated with hormone biosynthesis, conjugation and signaling. Of the 646 genes of the list (see Supplementary Data 5), 340 are differentially expressed for one or more time point in *rpn12a-236* at *p* < 0.0001. After hierarchical clustering, the dataset was divided in to 8 clusters, which are presented on the right. Average *Z*-score was plotted overtime for each cluster. **c** Pie charts illustrating the distribution of genes involved in hormone signaling among the clusters generated in **b**. ABA: abscisic acid, CKs: cytokinins, ET: ethylene, IAA: auxin, SA: salicylic acid.

Firstly, we noticed that the expression level of nearly all 26S proteasome subunits was significantly higher in the *rpn12a-236* mutant irrespective of the IDL treatment (Fig. S7a and Supplementary Data 2), which also correlated to an enhanced protein accumulation (Fig. S7b). Such feedback regulation on the expression of the proteasome subunits has already been observed and appears as a common feature in proteasome mutants^12,22^. Secondly, we established a manually curated list of 169 senescence-responsive genes and checked their expression profile across the time-course in IDLs (Fig. 5a and Supplementary Data 3). After a hierarchical clustering based on a Pearson correlation, 5 main clusters were defined. Clusters 1 and 4 contained genes that responded quickly to the darkening treatment as transcription was rapidly induced in both genotypes; however, not to the same amplitude. Cluster 1 included several senescence-associated genes (SAGs) known to have their product regulated by the proteasome: such as the E3 ligase *AIP2*, the MYC2-regulator *JAZ3*, the PROTEASOME REGULATOR1 (*PTRE1*), the ABA-responsive gene (*BT2*), or the transcription factor ETHYLENE-INSENSITIVE3 (*EIN3*). Cluster 4, which contained genes with significantly higher transcript abundance in WT than in *rpn12a-236* also contained well-known SAGs (*ORE1*, *SAG12*, *SAG18*, *NYC1*, *NYE1*), ET-associated regulators (*EIN2* and *EBFs*), and other hormone-related transcripts from the IAA, cytokinins (CKs) and ABA biosynthesis and signaling pathways.

Notably, the ABA-responsive gene *BT2* present in cluster 1 was highly expressed in *rpn12a-236* while both *AtBHLH093* (cluster 4) and *AtSAG18* (cluster 4) were downregulated (Fig. 5a and Fig. S8). It was shown that the ortholog of *BT2* in *Malus domestica, MdBT2*, negatively affects *MdBHLH093*, a direct regulator of *SAG18*, through the action of the proteasome^23^. Thus, we hypothesized that a defect in the 19S particle may lead to a feedback loop modulating the expression of *AtBHLH093* (Fig. S8).

Thirdly, we compared the expression profile of genes involved in hormone biosynthesis, homeostasis and signaling, and which were supposedly associated with the delay of senescence in *rpn12a-236* IDLs. A manually curated list of 646 genes was created, of which 340 genes were differentially expressed (*p* < 0.0001) at least for one time point in the *rpn12a-236* mutant as compared to WT (Fig. S9 and Supplementary Data 4). A Pearson correlation-based hierarchical clustering analysis was performed, and 8 clusters were defined. The five first clusters exhibited a major difference between *rpn12a-236* and WT across the time-course (Fig. 5b) while clusters 6, 7, and 8 only differed at one time point (6 h or 3 days). Cluster 4 included 74 transcripts that were induced during the progress of senescence in WT but not in the mutant. This cluster contained transcripts encoding 15 ET response factors (ERFs), 2 CKs degradation (CKX2, CKX5) and 2 CKs conjugation enzymes (UGT73C5, UGT85A1), 6 transcripts belonging to the ABA and JA biosynthesis pathway (AAOs and OPRs), 10 SA signaling genes, as well as 5 IAA responsive genes. Interestingly, this cluster contained the transcripts encoding EBFs and EIN2 involved in the proteasome-dependent degradation of the EIN3 senescence-responsive transcription factor. However, as earlier noted *EIN3* was not differentially expressed between the two genotypes, whereas *ORE1*, a master regulator of senescence and direct target of EIN3, was significantly repressed in the mutant. This suggests a post-translational regulation-only of EIN3 in a mutant background. In addition, we checked the distribution of hormonal signaling DEGs within the 8 clusters. Most ET signaling genes were found in cluster 4 but also in clusters 6 and 7, and mostly showed an early reduction in transcript abundance (1 day, 3 day) in the mutant (Fig. 5c). SA and IAA signaling genes were also mostly downregulated in the mutant as they are found in clusters 4, 7 and 8. ABA-related DEGs were mostly downregulated at 3 days in cluster 6. Interestingly, CKs signaling genes were almost evenly distributed between clusters 4, 5, 6, 7 and 8, indicating a complex and temporal regulation in response to darkness. Taken together, the regulation of many genes involved in hormone metabolism were affected in the *rpn12a-236* mutant, suggesting a profound alteration in the typical hormonal homeostasis associated with the progression of leaf senescence.

### RPN12a is necessary to maintain Arabidopsis hormonal balance during DIS

**Fig. 6:**
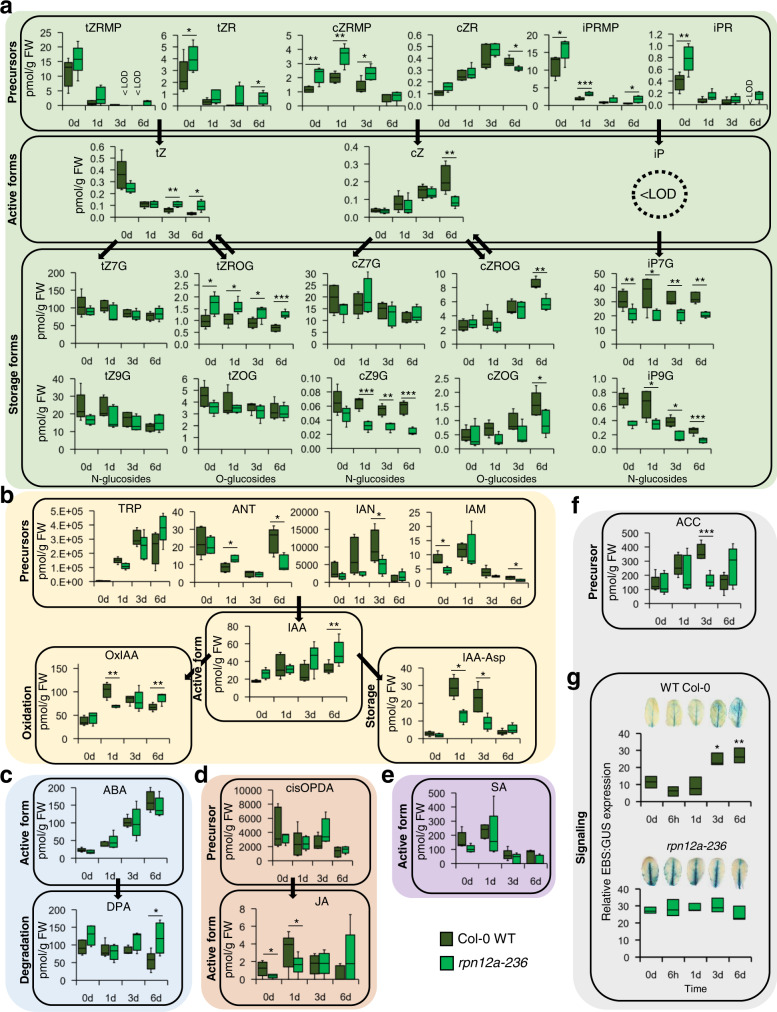
Phytohormones content in *rpn12a-236* and WT Col-0 individually darkened leaves. Total pools of selected metabolites related to **a** cytokinins, **b** auxins, **c** abscisic acid, **d** jasmonic acid, **e** salicylic acid, and **f** ethylene were quantified by LC-MS (*n* = 5) (for details, see Supplementary Data 5). **g** Effect of darkening on the expression of the ethylene reporter *EBS:GUS* in WT Col-0 and *rpn12a-236*. The pictures are representative of 3 independent biological replicates of leaves examined for each time point. Relative expression was quantified using ImageJ and error bars represent the standard deviation. Statistics: time effect. The interquartile range for the box plots is from the 25th to the 75th percentiles, and the middle line is the median (*n* > 4 biologically independent replicates); a Student’s *t*-test (two sided) showed statistically significant differences at *p < 0.05, **p < 0.01, ***p < 0.001. <LOD = not detected.

To cross-validate the transcriptomic data, a broad hormone analysis (“hormonomics”) was carried out with the WT and *rpn12a-236* under IDL. In brief, a LC/MS-based method allowed for the simultaneous detection of many classes of phytohormones as well as their precursors^24^. Twenty metabolites related to CKs, seven to auxin, two to JA, one to SA, and two to ABA were quantified and many significant differences between the genotypes were observed (Supplementary Data 5). For CKs, trans-zeatin precursors (tZRMP and tZR), active (tZ) and storage (tZROG) compounds accumulated strongly in the mutant, but the cis-zeatin (cZ) pathway that was significantly less abundant (Fig. 6a), leading to an imbalance in the tZ/cZ ratio in *rpn12a-236* during IDL (Fig. S10). The active form isopentenyladenine (iP) was not detected in our experiment at any time point, but a strong increase of precursors and decrease in N-glucoside compounds was observed in *rpn12a-236* (Fig. S11). Although the auxin precursors content seemed lower in *rpn12a-236*, IAA concentration was overall higher in the mutant than in WT–although only significant at 6 days (Fig. 6b). On the other hand, the storage conjugated form, IAA-Asp, was found more abundantly in IDLs of WT after 1 day and 3 days. Similarly, the oxidized form of IAA, 2-oxindole-3-acetic acid (OxIAA), was significantly more abundant in WT after 1 day IDL, whereas it accumulated in the mutant at a later time point (i.e., 6 days), suggesting that the IAA degradation pathway was more active during the initiation of senescence in WT (Fig. 6b and Fig. S12). In response to the IDL treatment, ABA massively accumulated in both WT and mutant lines (ca. a 10-fold changes); however, a strong accumulation (2-fold) of dihydrophaseic acid (DPA), an inactive form derived from the degradation of ABA, was observed in *rpn12a-236* at 6 days of IDL (Fig. 6c), suggesting a specific regulation in the proteasome mutant. JA accumulated quickly upon darkening in the WT, but this appeared delayed in the mutant, as it peaked only after 6 days (Fig. 6d). No significant changes in SA were observed in *rpn12a-236* (Fig. 6e). ET cannot be detected by our LC/MS method, but we quantified 1-aminocyclopropane-1-carboxylate (ACC) measurement, a precursor of ET^25^. ACC was significantly higher in WT after 3 days of treatment (Fig. 6f). Furthermore, we checked the downstream signaling pathway using the ethylene-inducible EBS:GUS reporter transgene introduced by crossing into the *rpn12a-236* background. This construct consists of a GUS reporter gene whose expression is driven by a synthetic EIN3-responsive promoter^26^. Although the GUS signal was stronger in the *rpn12a-236* mutant in IDL at similar growth stage, it was mostly localized to the leaf midrib in both genotypes at early time points. However, after 3 and 6 days of darkening, the signal was seen in mesophyll tissue of the WT but not in the IDLs of *rpn12a-236* (Fig. 6g). Image-based quantification of the signal confirmed these visual observations. Therefore, we concluded that the EIN3-responsive promoter expression is independent of the IDL treatment in *rpn12a-236* in contrast to the WT. Taken together, hormone metabolism was modified in *rpn12a-236* in a manner consistent with what could be expected from a mutation affecting dark-induced senescence in individually darkened leaves.

## Discussion

Based on our investigation with an EMS mutant named *rpn12a-236*, we show that RPN12a—an ortholog of yeast and human RPN12—influences both the vegetative growth and reproductive phase of plants (Fig. 2), the latter generally being associated with leaf senescence in annual plants. Smalle and co-workers reported a *rpn12a-1* mutant in Arabidopsis^15^, which exhibited several mild growth defects as well as an insensitive response to kinetin, suggesting a potential perturbation of the CK-dependent signaling. Yet, no further investigations have been performed to clarify the hormonal regulations associated with a defective proteasome. Furthermore, the authors also evidenced that both the full-length *RPN12a* RNA and a chimeric *RPN12a-NPTII* RNA were produced in this T-DNA insertion mutant. The *rpn12a-1* exhibited only a reduced 26S activity, presumably resulting from the competition between the native and the chimeric forms of the protein. In our study, the presence of a premature stop codon led to a truncated C-terminal version of RPN12a, however aside from the Whinged Helix domain. Highly conserved C‐termini are generally associated with specific functions, and it has been shown in yeast that the C-terminal region of RPN12 is necessary for the incorporation of the protein into the lid^27^. Thus, we can confidently propose that the truncated portion in *rpn12a-236* contains a docking signal necessary for this subunit to bind to one or several other member(s) of the proteasome in Arabidopsis. Furthermore, the reduced 26S proteasome accumulation in *rpn12a-236* when running native-PAGE indicates that either the 26S proteasome is not stable or the 19S RP interaction with the 20S CP is flawed (Fig. 3b). In either case, such structural defects in the 26S negatively affects the activity of the proteasome. While this led to an accumulation of the Ubn–protein conjugates in *rpn12a-236* (Fig. 3a), it also increased the chymotrypsin-like activity associated with a higher 20S CP pool, which indicates an increased degradation of the non-ubiquitinated proteins. Interestingly, it was previously observed that oxidized proteins are targets of the 20S particle^28^. ROS production, which is exacerbated during cell death events^29–34^, can lead to several post-translational modifications including lipoxidation, carbonylation, glycoxidation, and oxidation of aromatic- and sulfur-containing moieties^35^. Since mutations in the 19S particle induced an enhanced non-ubiquitin-dependent protein degradation via an exacerbated 20S activity^11^, we hypothesized that *rpn12a-236* would have a lower level of carbonylated proteins, which in turn could partially explain the functional stay-green phenotype of this mutant. This was indeed confirmed, as the level of carbonylated proteins was stable in *rpn12a-236* during the darkening treatment, whereas it increased in WT over the progression of senescence (Fig. S5). Moreover, the higher level of carbonylated proteins in WT can also be partly explained by the higher level of H_2_O_2_. It is known that during the progression of leaf senescence H_2_O_2_ accumulates^36^. Interestingly, it was reported that an excess of ROS induces a protein aggregation that clogs up the proteasome^37^. Such congestion of the ubiquitin proteasome system activity can lead to the accumulation of insoluble proteins, which causes the induction of PCD as earlier reported in hybrid tobacco cells^38^. Hence, while ROS accumulation contributes to leaf senescence during IDL in Arabidopsis WT plants, the accumulation of the 20S particle would promote an exacerbated degradation of the protein aggregates, thus protecting the leaf from cell death. Even though ROS are taking part to the signaling mechanisms triggering the induction of cell death^39^, we cannot rule out the fact that the oxidative stress is lower in response to darkness as a consequence of the non-induction of senescence rather than a cause for this non-induction. Nonetheless, the accumulation of ROS is also strongly influenced by hormones, especially ET; and both ROS and ET regulate the senescence-associated cell death during plant stress response^40,41^. Yet, the hormonal homeostasis regulating the onset and progression of leaf senescence is complex and involves additional phytohormones. ABA, ET, JA, strigolactone and SA are known positive regulators of senescence, while IAA, brassinosteroids, and CKs delay the process^42–49^. Furthermore, as the action of these hormones is transient, they require fine-tuned coordination between the biosynthetic, signaling, and degradation pathways associated with these hormones. A strong relationship ties hormone biosynthesis and signaling (including their catabolism) with the 26S proteasome, as this orchestrates the plant’s developmental program.

**Fig. 7:**
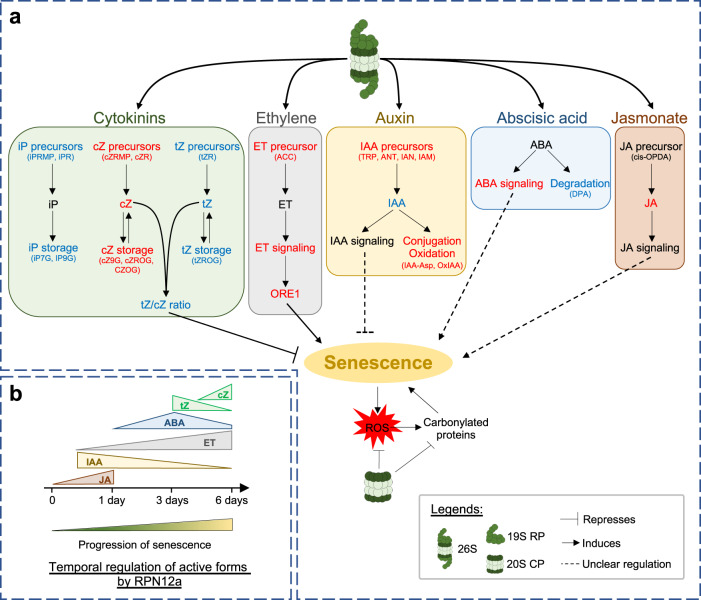
RPN12a-dependent regulation of senescence-associated cell death in leaves. **a** In IDLs, via the RPN12a structural component of the 19S RP, the 26S proteasome greatly impacts the cytokinins, ethylene, abscisic acid and auxin biosynthesis and signaling pathways. For instance, the tZ/cZ ratio decreases gradually leading to a reduction of the senescence-repressing tZ activity. IAA levels are also reduced possibly due to an increase of conjugated and oxidized IAA levels. The ET pathway is strongly enhanced by the proteasome activity, which leads to the induction of many catabolic processes via ORE1 and the SAGs, and primarily targeting the degradation of chloroplasts. The ABA degradation pathway (DPA) is lowered thus potentially increasing the ABA availability. JA concentration is particularly enhanced during the early stages of darkening perceived by the plant. The 20S CP can modulate the level of senescence-derived ROS, which are known to enhance the level of carbonylated proteins over time. In blue: negatively influenced; and in red: positively influenced, by the 26S proteasome during leaf senescence; **b** Temporal component for the regulation of hormone active forms by RPN12a.

Although a link between the 26S proteasome and senescence-associated cell death is commonly accepted, the effect of a dysfunctional 26S proteasome on the induction and progression of leaf senescence has remained unexplored at the molecular level. Here, we demonstrate that the lack of RPN12a leads to a specific and transient modulation in the metabolism of a subset of hormones, which contributes to the homeostasis controlling the induction of cell death in a leaf under stress (Fig. 7a). Indeed, the combined transcriptome and hormonal profiling indicates that RPN12a impacts several active forms of hormones (Fig. 7b), and subsequently hormone signaling related genes during DIS (Figs. 4c and  5b). JA is among the first RPN12a-dependent hormonal pathways to be affected (Figs. 6d and  7). This translates into an induction of several JAZs, potentially triggering the degradation of the MYC2 transcription factor by the proteasome. The loss of RPN12a function strongly delays this JA production. After 1 day, RPN12a durably affects the IAA pool by either lowering its production or increasing conjugation and degradation (Figs. 6b and 7). At this stage, it is tempting to speculate about a direct effect of the cell death-dependent accumulation of ROS onto the OxIAA levels, a mechanism proposed to occur in root tips^50^. In parallel, ET precursors and signaling genes are strongly induced in a RPN12a-dependent manner (Figs. 5a and 6f, g). ABA accumulates during DIS, and its degradation pathway (via DPA) decreases at later stages of the cell death progression (Fig. 6c). While ABA can positively regulate the progression of senescence via a succession of repressions (BT2, which in turn represses BHLH093 a transcription factor positively regulating *SAG18*^23^ (Fig. S8), the fact that *BT2* was upregulated in *rpn12a-236* (Fig. 5a) suggests that an additional regulatory mechanism is involved, such as the sugar status in cells as it was reported in Arabidopsis^51^. CKs are crucial to the progression of senescence, and the ratio of tZ/cZ is particularly affected during the latest stage of senescence (Fig. 6a and Fig. S10). Interestingly, the expression of *CKX* genes is mostly repressed in the mutant suggesting an inhibition of the CKs degradation. Furthermore, reversible O-glucoside storage compounds accumulated differentially in *rpn12a-236*. cZ O-glucosides were found in significantly lower amount than in WT, whereas an accumulation of tZROG was observed in the mutant. Thus, we hypothesized that reversible storage compounds can feed the tZ pool in *rpn12a-236* (Fig. 7). For catabolites defined as irreversible, N-glucosides (i.e., tZNGs, cZNGs, iPNGs)) were proposed to have a direct anti-senescent effect^52^. Yet, this contrasts with a recent study, which proposes that tZNGs could be converted back to tZ within minutes of exogenous application^53^. Therefore, although the tZ pathway is clearly promoted in *rpn12a-236*, we cannot confidently speculate on whether the RPN12a-dependent modulation of the N-glucosides levels may influence the progression of leaf senescence.

To conclude, members of the plant and animal kingdoms undergo multiple cell death events during their developmental programs. Yet, despite well conserved structure and functionality, it appears that the 26S proteasome differentially regulates the progression of cell death in these distinct taxonomic clades. Interestingly, several studies showed that the overexpression of certain subunits of the proteasome could extend the lifespan of organisms. For example, overexpression of the β5 subunit of the 20S or of the RPN6 subunit of the 19S proteosome increased lifespan of *Caenorhabditis elegans* under certain conditions^54,55^. Similarly, overexpression of RPN11 counteracted senescence in *Drosophila melanogaster*^56^. In Arabidopsis, overexpressing the *RPN5a* gene induced premature senescence among other developmental phenotypes in Arabidopsis^57^. The specificity of the proteasome Ub-dependent degradation pathway is achieved by the E3 ligases and the 19S ubiquitin receptors, namely RPN1, RPN10 and RPN13, that recognize specific lysine chain types^58^. RPN12 is considered a structural subunit of the 19S proteasome and is the last member to be assembled in the lid, which triggers the conformational remodeling of the lid to join the base^27,59^. Intriguingly, it was shown in yeast that the base RPN10 and the lid RPN12 subunits interact^60^. Therefore, it is conceivable that the specific response observed in the *rpn12a-236* mutant could be due to its interaction with RPN10, an assumption further supported by the fact that detached leaves placed in darkness exhibited a delayed senescence phenotype in *rpn10-1* and *rpn10-2* mutants as compared to wild type (WT) plants^12,61^. Therefore, future work should investigate in detail whether additional subunits of the 19S are also involved in the regulation of cell death and to which extent these responses overlap. Also, to our surprise, the *rpn12a-236* mutant exhibited an accelerated chlorophyll loss during developmental senescence when assessed from the Boyes 5.10 developmental stage. If assessed from the days after sowing, *rpn12a-236* would be considered as a mutant with a delayed developmental senescence. This discrepancy likely originates from the ubiquitous effect of an altered RP on both vegetative and reproductive phases of the plant. Indeed, it is plausible that an impaired 19S also influences some regulatory feedback loops in the flowering process, which in turn would influence the progression of leaf senescence when the plant goes from vegetative to reproductive stages. Yet, additional investigations should be conducted to understand the differential impact of RPN12a on developmental senescence. Combined with biochemical studies and analysis of tag recognition, this will ultimately determine how the 26S proteasome can modulate cell death and provide suitable targets for bioengineering-based strategies aiming at improving crop resistance and perhaps also provide alternative approaches to counteract certain cancer and neurodegenerative diseases in humans.

## Methods

### Plant material and growth conditions

*Arabidopsis thaliana* Columbia-0 (Col-0) WT and mutant plants were grown under short-day photoperiod (SD: light 8 h at 22 °C, dark 16 h at 17 °C) at 65% relative humidity and 180 μmol m^–2^ s^–1^ photosynthetically active radiation (PAR) or long-day photoperiod (LD: light 16 h at 22 °C, dark 8 h at 17 °C) at 65% relative humidity and 150 μmol m^–2^ s^–1^ PAR on a mixture soil:vermiculite 3:1.

For SD grown plants, at 5–7 weeks after sowing, two leaves of each plant were covered individually for IDL^13^. Time of treatment is indicated in figures and legends. LD growth condition was used for seeds propagation.

For detached rosette leaves experiment, leaves from three independent randomized plants were incubated in a 3 mM MES solution for 7 days in darkness at room temperature.

For developmental senescence, rosettes from three randomized replicates were collected every 7 days after the first flower bud is visible, corresponding to the 5.10 Arabidopsis growth^16^.

Skotomorphogenesis experiments were done as described by Smalle and co-workers^15^. Sterilized and stratified seeds were sown on Gamborg’s B-5 medium. Plates were covered with aluminum foils and transferred in a LD growth room. Seedlings were observed after 10 days treatment.

### EMS mutagenesis, screening, and sequencing

The *rpn12a-236* mutant was identified by screening approximately 6000 EMS (Ethyl-Methan-Sulfonate)—mutagenized WT plants (purchased at the INRA-Versailles Arabidopsis stock Center; France). Plants were grown for 7 weeks under SD conditions and leaf senescence was induced by darkening 2 leaves for 6 days. Plants with covered leaves that remained green were kept and M3 plants were screened for the same phenotype. The mutant plants were backcrossed two times (BC2) to wild-type (WT) Col-0 plants and used for all analysis.

To identify the causal mutation, whole genome sequencing (WGS) was used^62^. DNA from 8 BC2F3 *rpn12a-236* stay-green plants and 4 WT plants was extracted using a CTAB protocol. Frozen leaves (one leaf per tube) were grinded using steal beads, 500 μl of CTAB buffer (2% cetyl trimethylammonium bromide, 1% polyvinyl pyrrolidone 40, 100 mM Tris-HCl (pH 8), 1.4 M NaCl, 20 mM EDTA) was added to the powder and tubes were vortexed. Samples were incubated for 30 min at 60 °C and centrifuged for 5 min at 14,000 × *g*. The liquid phase was transferred in a new tube and samples were treated with 5 μl of RNAse A (10 mg/ml) for 20 min at room temperature. One volume of chloroform:IAA (24:1) was added and tubes were vortexed for 5 s. Tubes were centrifuged for 1 min at 14,000 × *g* and upper phase was carefully transferred in a new tube. For precipitation, 0.7 volume of cold isopropanol was added and left at −20 °C for at least 15 min. Tubes were centrifuged for 10 min at 14,000 × *g*, supernatant was discarded, and pellets were washed using Cold EtOH 70%. After tubes inversion, tubes were centrifuged for 5 min at 14,000 × *g*, supernatant was discarded, and pellet was air dried for 20 min. Pellet rehydration is made using 50 to 100 μl of TE. DNA integrity was assessed by gel electrophoresis, concentration was assessed by Qubit fluorometric quantification (Life technologies, Q32866) and purity by ND-2000 (NanoDrop technologies, Inc, Wilmington, DE, USA). Same DNA quantity was pooled to reach 6 μg total DNA amount in 100 μl. Finally, DNA was pelleted using a Speed-Vac. DNA sequencing was performed at Novogene Technology Co., Ltd (Animal and Plant Resequencing (WGS)).

### Plasmid construction and Agrobacterium-mediated transformation

cDNA of *RPN12a* was synthesized and amplified from WT RNA using primers containing attB recombination sites for Gateway cloning Rpn12a_GTW-F and Rpn12a_GTW_S-R. PCR product was cloned in the pDONR207 donor vector by BP cloning and insert was transferred in pGWB2 (35S promoter, no tag) and pUB-DEST (UBQ10 promoter, no tag) destination vectors.

The *RPN12a* genomic locus (861 bp prior to the ATG and 440 bp after the stop codon) was amplified from WT DNA using primers containing attB recombination sites for Gateway cloning Rpn12a-P_GTW-F and Rpn12a-T_GTW-R. PCR product was cloned in pDONR207 through BP reaction and inserted in pGWB1 (no promoter, no tag) destination vector.

Plasmids were used for *Agrobacterium tumefaciens* (GV3101::pMP90) transformation of *rpn12a-236* mutants using the floral dip method. In vitro selections of plantlets were made on ½ MS + 0.1% sucrose media containing Kanamycin (50 μg/ml, T-DNA from pGWB1 and pGWB2) or Basta (10 μg/ml, T-DNA from pUB-DEST). Primers are listed in Supplementary Data 6.

### RPN12a C-terminal conservation

For sequence conservation of RPN12a C-terminal part, Weblogo^63^ was used to visualize sequence conservation in 187 C-terminal RPN12a-like domains in *Viridiplantae*. This was done through alignment with the last 44 aa of RPN12a (containing the mutation in *rpn12a-236*). The height of each letter (score on *y*-axis) indicates the relative frequency of an amino acid at a position.

### Chlorophyll measurements

Leaves were sampled in tubes containing steal beads and frozen using liquid nitrogen. In all, 10–50 mg of powder was measured and transferred directly in tared 80% acetone tube for chlorophyll extraction. Samples were vortexed 30s and incubated for 15 min in darkness. Samples were vortexed again and centrifuged at 15,000 × *g* for 10 min at 4 °C. Absorbance for the supernatant was measured at 750 nm for the background, at 663 nm for chlorophyll *a* and at 647 nm for chlorophyll *b* with a spectrophotometer. The chlorophyll content was calculated using the following formula^64^: Chl_*a*+*b*_ = 7.15(*A*_663_ – *A*_750_) + 18.71(*A*_647 _– *A*_750_) (μg/ml).

### SDS-Page immunoblots

For immunoblot analysis, Arabidopsis total protein extracts were prepared using a protein extraction buffer (100 mM Tris-HCl pH 7.5, 50 mM EDTA, 250 mM NaCl, 0.05% SDS). Protein quantification was done with a Bradford protein assay (Bio-Rad, Protein Assay Dye Reagent Concentrate, 500-006). Extracts were mixed with Laemmli sampling buffer (Bio-Rad, 4x Laemmli Sample Buffer, 1610747) supplemented with 10% β-mercaptoethanol and incubated at 95 °C for 10 min before separating the protein mixtures on reducing 12% polyacrylamide gel.

After migration at 100 V, proteins were transferred during 1 h at 270 mA onto a 0.45 µm nitrocellulose membrane. Blot was blocked with 5% milk in TBS-T for 1 h following by an overnight incubation at 4 °C with the specific polyclonal primary anti-RPN12a antibody (1/1000, Abcam, ab98959), the anti-Ubiquitin antibody (1/1000 in TBS-T, Sigma-Aldrich, U5379), the anti-PBA1 antibody (1/3000 in TBS-T, Agrisera, AS19 4260) or the anti-RPN6 antibody (1/2000 in TBS-T, Agrisera, AS15 2832A) diluted in 2% milk in TBS-T. After 1 h incubation at room temperature with a goat anti-rabbit secondary antibody conjugated to horseradish peroxidase (1/10000 in 2% milk in TBS-T, Agrisera, AS09 602), visualization was carried out using a chemiluminescence kit (Agrisera ECL kit bright; AS16 ECL-N-100) and signals were detected using Azure c600 Western Blot Imaging system (Azure biosystems). Exposure time was 1–3 min. Signals were quantified using the ImageJ software (https://imagej.nih.gov/ij/).

### Protein purification

Proteins for the native-PAGE and the activity assay were prepared as previously described^11^. Briefly, thirty 10-day-old seedlings were frozen, ground and homogenized in 1.25 ml/g fresh weight proteasome extraction buffer (PEB: 50 mM potassium phosphate buffer pH 7.0, 2 mM MgCl_2_, 5% glycerol, 5 mM 2-mercaptoethanol and 10 mM ATP). The extract was clarified by centrifugation at 8000 × *g* for 15 min at 4 °C. The protein concentration of the supernatant was determined using a Bradford protein assay.

### Native-PAGE immunoblot

For separation of proteasome complexes, crude plant extracts were subjected to Blue native polyacrylamide gel electrophoresis (BN-PAGE)^65^. For native gel preparation, a 3.5–12% gradient (48% acrylamide, 1.5% bis-acrylamide) of running gel (in 50 mM bis-tris (pH 7), 500 mM 6-aminocaproic acid buffer) was casted in 1 mm cassette using a Gradient Mixer and a peristatic pump (speed ≥ 200 rpm). Running ger topped with a 3% stacking gel (20% acrylamide, 5% bis-acrylamide in same gel buffer). Separate cathode (15 mM bis-tris, 50 mM tricine) and anode buffer (50 mM bis-tris, pH 7) was used for running the gel.

For sample preparation, 10 µg of total proteins was mixed gently with 1x ACA (100 mM BisTris-HCl, pH 7.0, 500 mM 6-aminocaproic acid, 0.25 mg/ml Pefa, 10 mM NaF, 1 mM EDTA) to reach a 10 µl final volume. One volume of 4% digitonin (in 100 mM BisTris-HCl, pH 7.0, 0.5 M 6-aminocaproic acid, 0.25 mg/ml Pefa, 10 mM NaF, 1 mM EDTA, 4% Digitonin) was added to the mix and the reaction was incubated for 8 min at 4 °C with gentle shaking (speed ≥ 200 rpm). Samples were centrifugated for 25 min at 4 °C at a speed of 14,000 x *g*. The supernatant was transferred to a new microcentrifuge tube and BN sample buffer (5% Serva Blue G, 100 mM BisTris-HCl, pH 7.0, 0.5 M 6-aminocaproic acid, 30% sucrose) was added at 10/1 sample to dye ratio. Gel was run in 4 °C at 75 V for 45 min, 100 V for 30 min and finally at 125 V until the dye front reached the bottom of the gel.

For 1st dimension transfer, proteins were transferred on a PVDF membrane overnight at 4 °C at 25 V. For 2nd dimension run and transfer, lines were cut from the 1st dimension, incubated with Laemmli buffer (with 0.2 % w/v SDS and 100 mM DTT) for 30 min at room temperature and ran onto a 12% SDS running gel (5% stacking gel) at 150 V for 1.5 h at room temperature and proteins were transferred on a nitrocellulose membrane overnight at 4 °C at 25 V. After transfer, membranes were blocked with 2% milk in TBS-T for an hour at RT and incubated for 3 h at RT with specific polyclonal primary anti-PBA1 antibody (1/3000, Agrisera, AS19 4260) or anti-RPN12A antibody diluted in blocking buffer. After 1 h incubation at room temperature with a goat anti-rabbit secondary antibody conjugated to horseradish peroxidase (1/10000, Agrisera) in blocking buffer. Signals were detected using Azure c600 Western Blot Imaging system with exposure time of 15–30 s.

### Chymotrypsin-like activity measurements

Chymotrypsin-like peptidase activity was measured using the fluorogenic peptide N-succinyl-Leu-Leu-Val-Tyr-7-amino-4-methylcoumarin (Suc-LLVY-AMC; Abcam, ab142120). For in vitro assay, 10 µl of protein extract were incubated with 90 µl of 50 µM Suc-LLVY-AMC, supplemented or not with 80 µM of MG132 proteasome inhibitor (Sigma-Aldrich, SML1135), at 37 °C for 20 min. To quench the reaction, 1 ml of 80 mM sodium acetate (pH4.3) was added. Released AMC was monitored according to the following excitation/emission wavelengths (380 nm/440 nm). Fluorescence of the reaction with MG132 is subtracted from the fluorescence without MG132 to obtain a relative fluorescence. For proteasome activity on native samples loaded on a first-dimension gel, membranes were incubated for 1 h in an activity buffer (50 mM Tris pH 7.5, 5 mM MgCl_2_, 10% glycerol, 1 mM ATP, 1 mM DTT, 0.1 mM Suc-LLVY-AMC). Exposure time at 365 nm was 15–30 s.

### Detection of oxidized proteins and hydrogen peroxide/superoxide

DNPH derivatization was done following the Oxyblot protein oxidation detection kit instructions (Merck Millipore, S7150).

Hydrogen peroxide was detected by 3,3′-Diaminobenzidine (DAB; Sigma-Aldrich, D12384). Leaves were sampled and placed into 5 cm diameter petri dishes containing 5 ml of DAB staining solution (1 mg/ml DAB in sterile water pH3.8 with 1 M HCl). Vacuum infiltration was applied 2 times for 5 min. Dishes were covered with aluminum foil and kept overnight at room temperature. Following incubation, DAB solution was replaced by pure EtOH for bleaching.

Superoxide was detected by Nitro blue tetrazolium (NBT; ThermoFischer, 34035). Leaves were sampled and placed into 5 cm diameter petri dishes containing 5 ml of NBT staining solution (0.2% NBT in 50 mM sodium phosphate buffer (pH 7.5)). Vacuum infiltration was applied 2 times for 5 min. Dishes were covered with aluminum foil and kept overnight at room temperature. Following incubation, NBT solution was replaced by pure EtOH for bleaching.

### RNA extraction for RNA-seq

WT and *rpn12a-236* plants were grown, and samples of leaf blades were collected at 8 am, for the 0 days, 1 day, 3 days, 6 days time points for IDL/light control treatments and at 2 pm, for the 6 h time point for IDL/light control treatments. For all conditions, three biological replicates were used, each of them being a pool of two leaves from independent plants. RNA was extracted from approximately 40 mg of frozen material using an EZNA plant RNA kit following provider recommendations (Omega Bio-tek, R6834-01). Concentration was assessed by Qubit fluorometric quantification and purity by ND-2000. RNA integrity was checked with a Bioanalyzer 2100 expert and RNA 6000 Nano kits (Agilent, Santa Clara, CA, USA). RNA sequencing was performed at Novogene Technology Co., Ltd (HiSeq platforms, paired-end 150 bp sequencing strategy).

### RNA-seq bioinformatics

The data pre-processing was performed following the guidelines described here: http://www.epigenesys.eu/en/protocols/bio-informatics/1283-guidelines-for-rna-seq-data-analysis. Briefly, the quality of the raw sequence data was assessed using FastQC (http://www.bioinformatics.babraham.ac.uk/projects/fastqc/), v0.11.4. Residual ribosomal RNA (rRNA) contamination was assessed and filtered using SortMeRNA^66^ (v2.1; settings --log --paired_in --fastx--sam --num_alignments 1) using the rRNA sequences provided with SortMeRNA (rfam-5s-database-id98.fasta, rfam-5.8s-database-id98.fasta, silva-arc-16s-database-id95.fasta, silva-bac-16s-database-id85.fasta, silva-euk-18s-database-id95.fasta, silva-arc-23s-database-id98.fasta, silva-bac-23s-database-id98.fasta and silva-euk-28s-database-id98.fasta). Data were then filtered to remove adapters and trimmed for quality using Trimmomatic^67^ (v0.36; settings TruSeq3-PE-2.fa:2:30:10 SLIDINGWINDOW:5:20 MINLEN:50). After both filtering steps, FastQC was run again to ensure that no technical artefacts were introduced. Read counts were obtained using kallisto^68^ (v0.43.0) with the parameters quant -b 100 --pseudobam -t 1 --rf-stranded and using the TAIR10 cDNA sequences as a reference (retrieved from the TAIR resource). An overview of the data, including raw and post-QC read counts and pseudo-alignment rates is given in Supplementary Data 8. The kallisto abundance values were imported into R (v3.3.2; R Core Team 2015) using the Bioconductor^69^ (v3.3) tximport package^70^ (v.1.2.0). For the data quality assessment (QA) and visualization, the read counts were normalized using a variance stabilizing transformation as implemented in DESeq2. The biological relevance of the data—e.g., biological replicates similarity—was assessed by Principal Component Analysis (PCA) and other visualizations (e.g., heatmaps), using custom R scripts. Statistical analysis of gene and transcript differential expression (DE) between conditions was performed in R using the Bioconductor DESeq2 package^71^ (v1.14.1), with the following model: ~Line*Hours to account for both the genotype (WT or *rpn12a-236*) and harvesting time (d0, h6, d1, d3, d6). FDR adjusted *p*-values were used to assess significance; a common threshold of 1% was used. DESeq was also run with following models: ~Line and ~Hours to compare gene expression at fixed time and fixed genotype, respectively.

Hierarchical clustering analysis was performed on either (i) a subset of genes that are significantly (adj. *p* < 0.0001) differentially expressed between WT and *rpn12a-236* for at least one time point or (ii) a list of genes extracted from bibliographic data using MeV (MultiExperiment Viewer) based on a Pearson correlation and average linkage clustering. Within hierarchical clusters, genes specific to a given cluster were submitted to gene ontology (GO) enrichment analysis. This was done using the new version of gProfiler^72^.

#### Building a biological network based on GO terms

A biological network was built using the Cytoscape (version 3.9.0^21^)—compatible plug-in ClueGO (version 2.5.8^20^). GO terms (Biological Process) from significantly (*p* < 0.001) differentially expressed genes in *rpn12a-236* versus WT. Enriched GO terms upregulated in *rpn12a-236* (palette of blues) or downregulated (palette of yellows) as compared to WT were clustered according to their functional categories. The following parameters were applied to generate the networks: min GO level = 1, max GO level = 8, number of genes per term = 3, and 3.0% genes per term for all clusters; the minimum percentage for a cluster to be significant was 60%, both GO fusion and GO group were applied, Kappa score was fixed at 0.4, with an initial group size of 1 and a sharing group percentage set at 50%. The statistical test used for enrichment/depletion was a two-sided hypergeometric test with a Bonferroni step down correction method applied. The size of a node is proportional to the number of genes contributing to this node, while the intensity of the color is proportional to the significance of it (the minimum for significance being *p* < 0.05); gray nodes = overrepresented in the gene subset but not enriched significantly. For day 1, 3 and 6, the subset of genes differentially regulated at day 0 was subtracted to highlight the functional categories associated with cell death and minimize the constitutive contribution of a non-functional proteasome.

### Hormones content and pharmacology

WT and *rpn12a-236* plants were grown in SD, and leaves were harvested. Samples of leaf blades were harvested at 8 am, for 0 days, 1 day, 3 days, 6 days for IDL/light control treatments and at 2 pm, for 6 h for IDL/light control treatments and frozen using liquid nitrogen. For all conditions, five biological replicates were used. In all, 10–20 mg of powder was measured, transferred in a new collection tube, and stored at −70 °C. For CKs, auxin, JA, SA, ABA, and ACC samples were analyzed as previously described^24,25^.

We estimated ET content using the WT EBS:GUS reporter line. This construct consists of 5 tandem repeats of the *EDF1* promoter, a target of EIN3^26^. The reporter transgene was introduced into the *rpn12a-236* mutant background by crossing and lines were selected on 50 μg/ml of hygromycin. Plants were grown in SD conditions and IDL was performed on 6–7 weeks old plants. Leaves were collected after at 0 days, 6 h, 1 day, 3 days, and 6 days of IDL treatment in petri dishes and GUS coloration solution (50 mM Phosphate buffer pH 7, 10 mM EDTA pH 8, 0.24% Triton X-100, 2 mM X-Gluc) was added. Tissues were vacuum infiltrated at 100 ppm in GUS solution (3 × 10 min) and incubated overnight at 37 °C. GUS solution was removed, and leaves were rinsed with water. EtOH 70% was added, and dishes were gently agitated until tissues bleached.

### Statistics and reproducibility

All source data for graphs and charts are provided in Supplementary Data 7. Statistical analyses conducted and sample size are defined in each figure’s legend.

### Reporting summary

Further information on research design is available in the Nature Research Reporting Summary linked to this article.

## Supplementary information

Supplementary Information

Description of Additional Supplementary Files

Supplementary Data 1

Supplementary Data 2

Supplementary Data 3

Supplementary Data 4

Supplementary Data 5

Supplementary Data 6

Supplementary Data 7

Supplementary Data 8

Reporting Summary

## Data Availability

Data are provided in Supplementals, or in public repositories. Raw RNA-seq data can be found at the Gene expression Omnibus under the accession GSE212121.
