# Supplementary material for: The RPN12a proteasome subunit is essential for the multiple hormonal homeostasis controlling the progression of leaf senescence
Source: Commun Biol. 2022 Sep 30;5:1043. doi: 10.1038/s42003-022-03998-2 (PMC9525688; doi:10.1038/s42003-022-03998-2)
Supplement: Supplementary file 1 — Supplementary Information [file 42003_2022_3998_MOESM1_ESM.pdf]

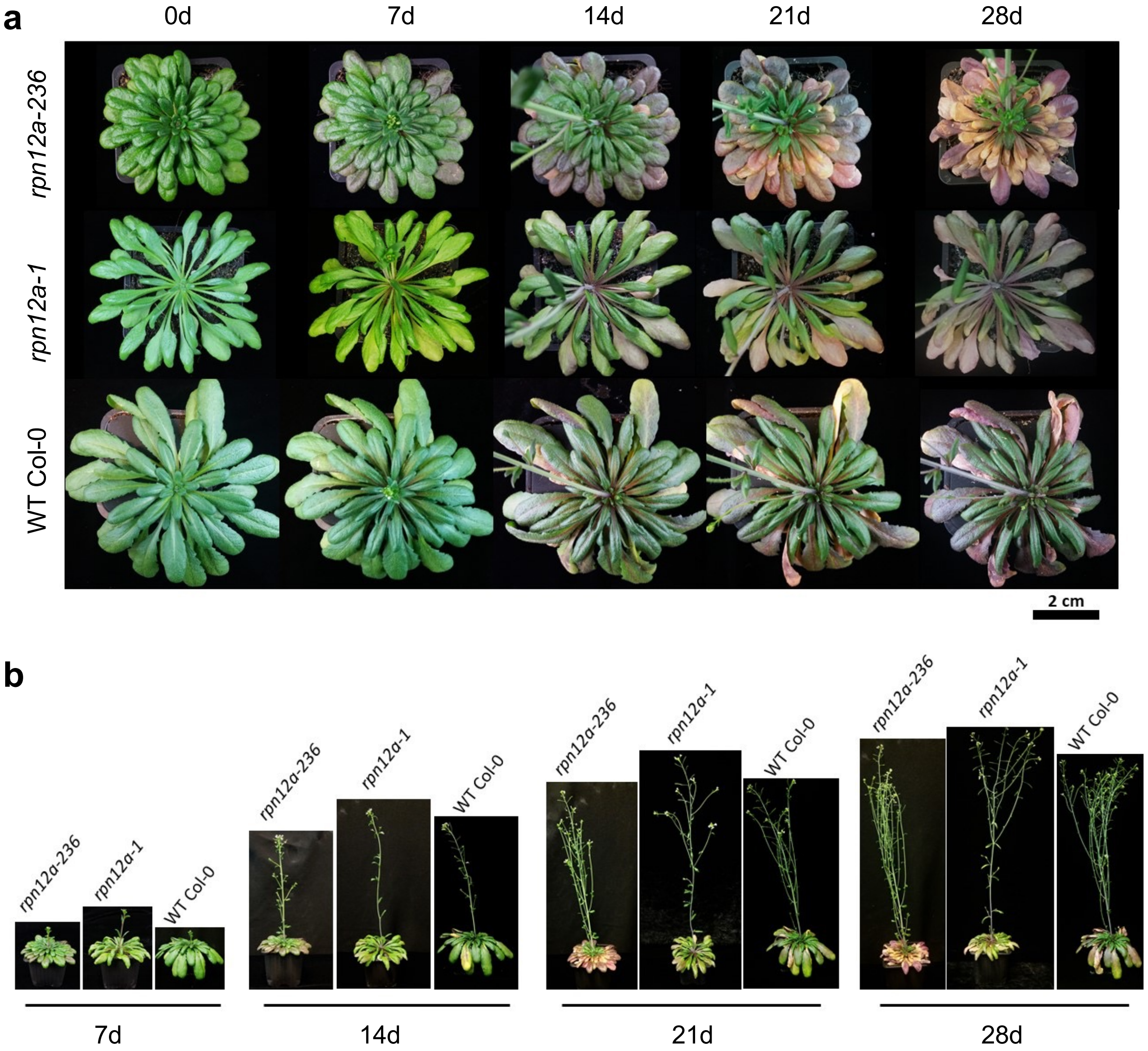

Figure S1. Comparison between WT and *rpn12a* mutants plants undergoing developmental senescence. Pictures were taken once a week after the first flower bud was visible (stage 5.10; Boyes et al., 2001). (**a**) Top view. (**b**) Front view.

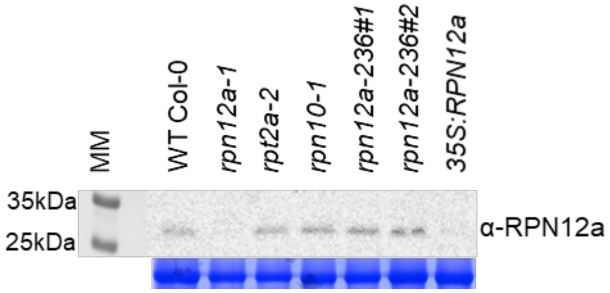

Figure S2. RPN12a immunoblot. Immunoblot analysis of total protein extracts from WT Col-0, *rpn12a-1*, *rpt2a-2*, *rpn10-1*, *rpn12a-236* and *35S:RPN12a* with antibodies raised against RPN12a. Loading control (Coomassie blue staining, focus on Rubisco large subunit) shows that equal amounts of proteins (10 µg) were loaded. MM = molecular mass marker. kDA = kilo Dalton.

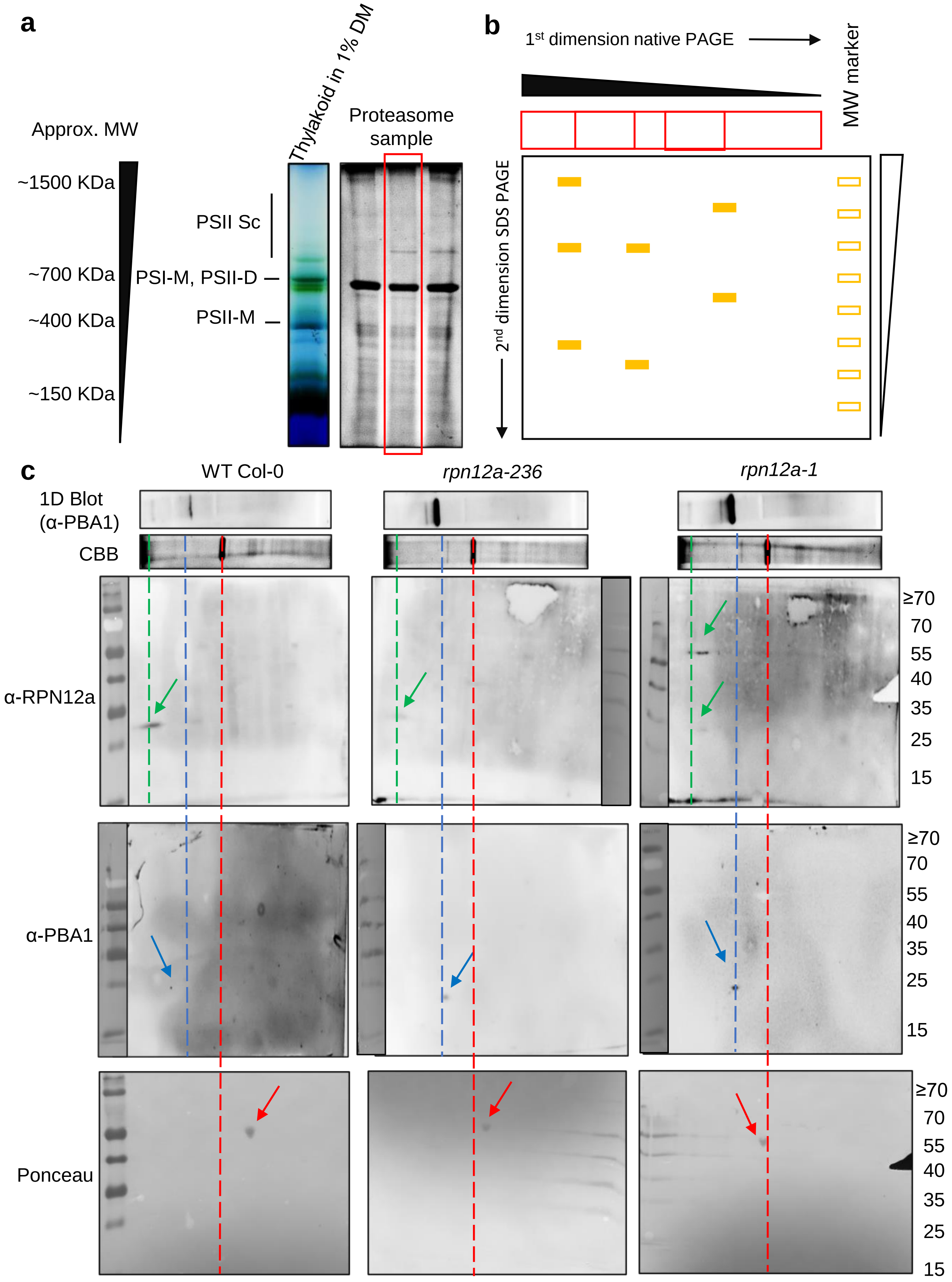

Figure S3. Second-dimension Native PAGE immunoblots. (**a**) The photosystem (PS) complexes and the proteasome samples were loaded on a 1D-gel. Molecular weight of the complexes were estimated by comparing with known molecular weights of *Arabidopsis thaliana* Col-0 photosynthetic pigment protein complexes separated under the same native conditions. (**b**) Schematic representation of a second-dimension run. (**c**) Native PAGE followed by a run on a second dimension SDS-PAGE on Col-0, *rpn12a-236* and *rpn12a-1*. Immunoblot analyses of the RPN12a subunit (top) PBA1 subunit (middle) were done on total protein extracts from 10-day-old seedlings. Ponceau staining are shown on the bottom. Green: 26S proteasome; Blue: 20S proteasome; red: proteasome-independent.

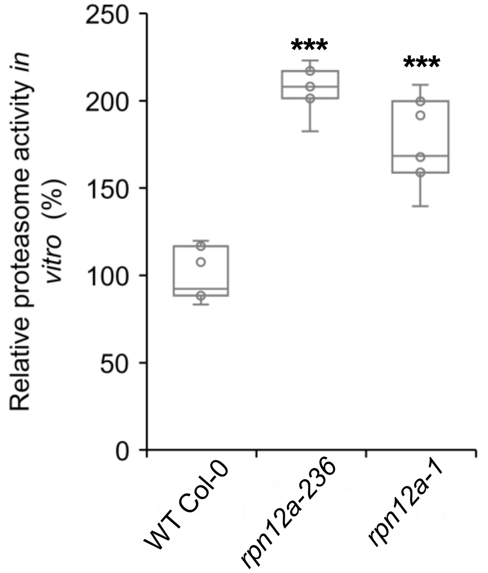

Figure S4. 20S Proteasome *in vitro* activity. *In vitro* activity test was performed in a UV microplate (n=7). Data are presented as corrected relative fluorescence normalized to the WT Col-0 level. The interquartile range of the box is from the 25th to 75th percentiles, and the middle line is the median; whiskers represent min and max values; a Student's t-test showed statistically significant differences at *** p<0.0001.

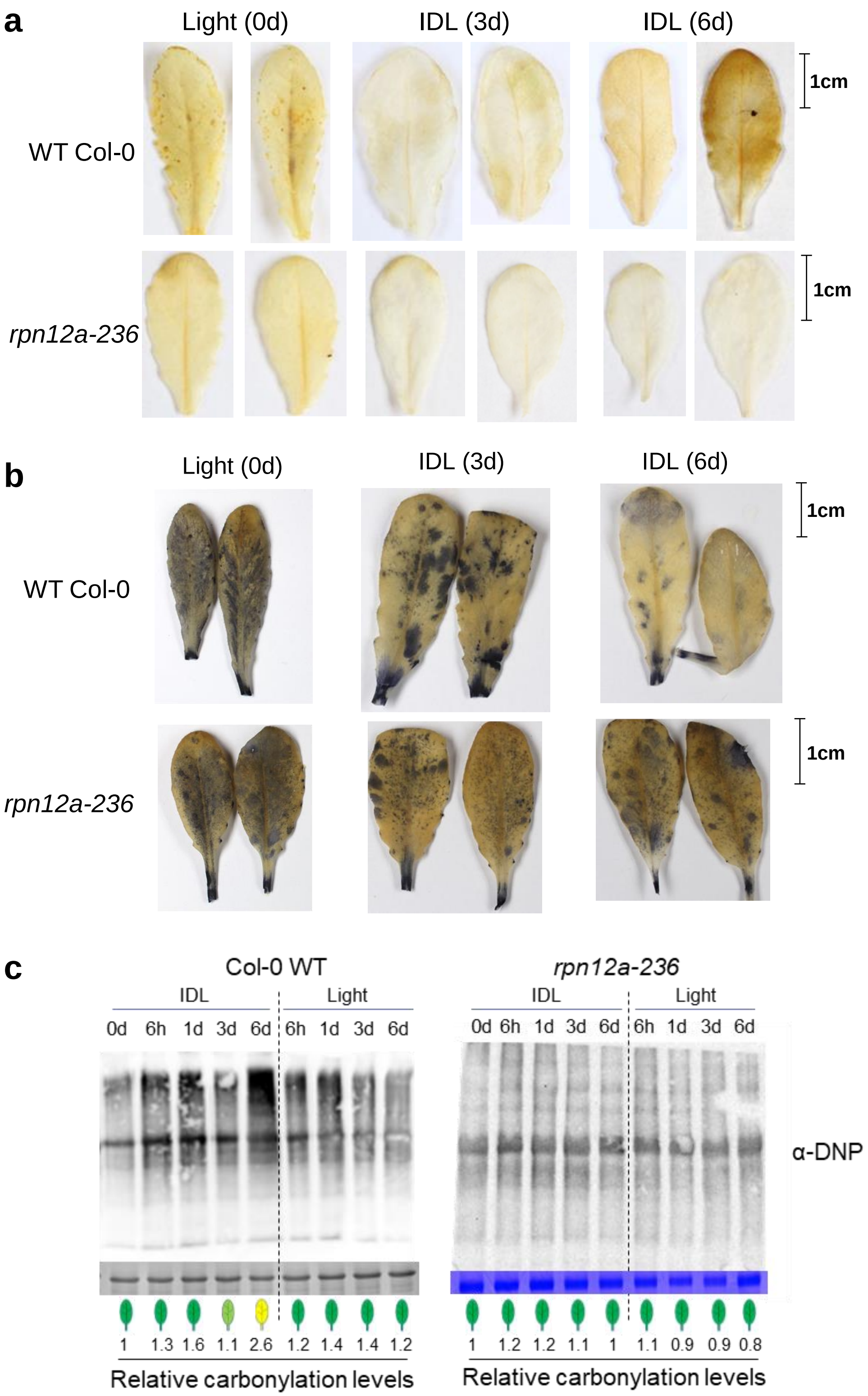

Figure S5. Levels of oxidation in a 19S proteasome mutant during DIS. (**a**) DAB coloration assay of H_2O_2 accumulation, and (**b**) NBT coloration assay of O_2^- accumulation in WT Col-0 and *rpn12a-236* mutant. IDL treatment was used to induce senescence on 6-week-old plants grown in SD for 0 day, 3 days, and 6 days. (**c**) Oxidized protein levels in Col-0 and *rpn12a-236* mutant. Equal amounts of total protein (20 µg) (Coomassie blue staining, focus on Rubisco large subunit) were subjected to SDS-PAGE and immunoblot analysis with anti-ubiquitin antibodies. Carbonylation levels were estimated, and fold-change compared to levels at the 0d time point (set to 1) was reported below the immunoblot. Proteins were extracted from 6- to 7-week-old SD grown plants treated with IDL or light for 0, 6h, 1 day, 3 days and 6 days. DNPH was derivatized by carbonyls and DNP levels were assessed by immunoblot anti-DNP

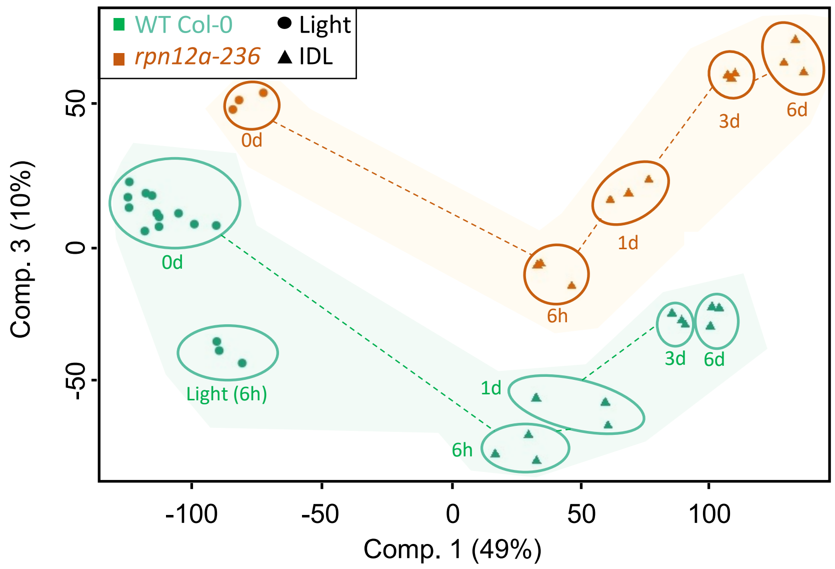

Figure S6. Principal Component Analysis from RNAseq data. Here, the first component (49%) versus third component (10%) are represented.

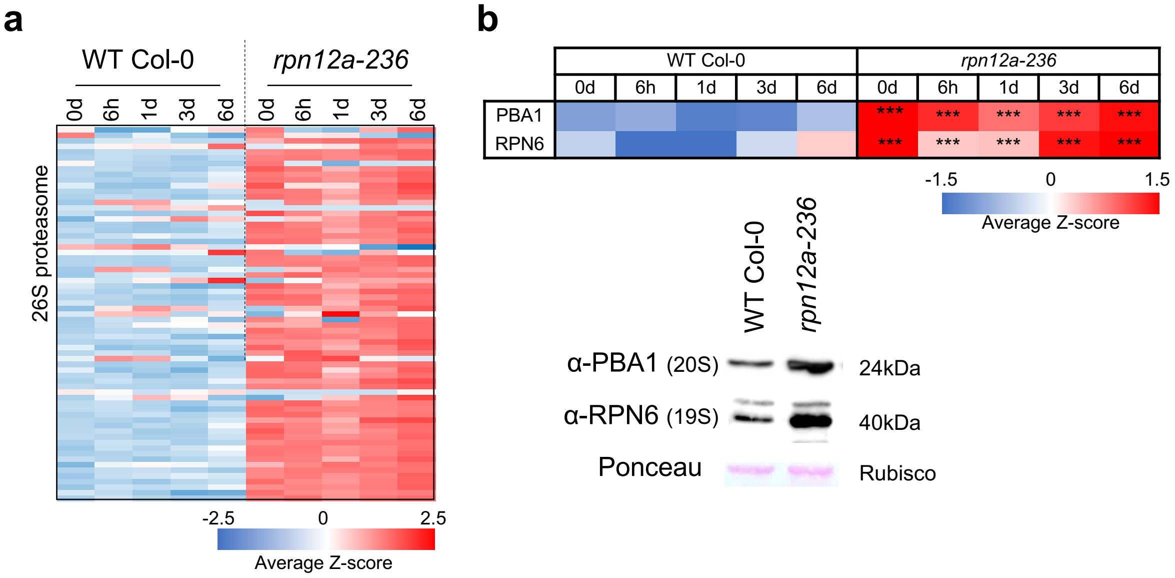

Figure S7. Regulation of the proteasome subunits. (**a**) Heatmap of proteasome subunits and interactants transcripts during DIS (n=66). (**b**) Focus on PBA1 (20S) and RPN6 (19S). Top: *PBA1* and *RPN6* expression was obtained from RNAseq experiment on Col-0 and *rpn12a-236*. The average Z-score is shown. A Student's t-test showed statistically significant differences from WT Col-0 at *** $p<0.0001$. Bottom: Immunoblots using PBA1 and RPN6 antibodies targeting the 20S and the 19S particles, respectively. 10 µg of total proteins, extracted from 6 week-old leaves of Col-0 and *rpn12a-236* grown in short day condition, were used. A ponceau staining was added as a loading control.

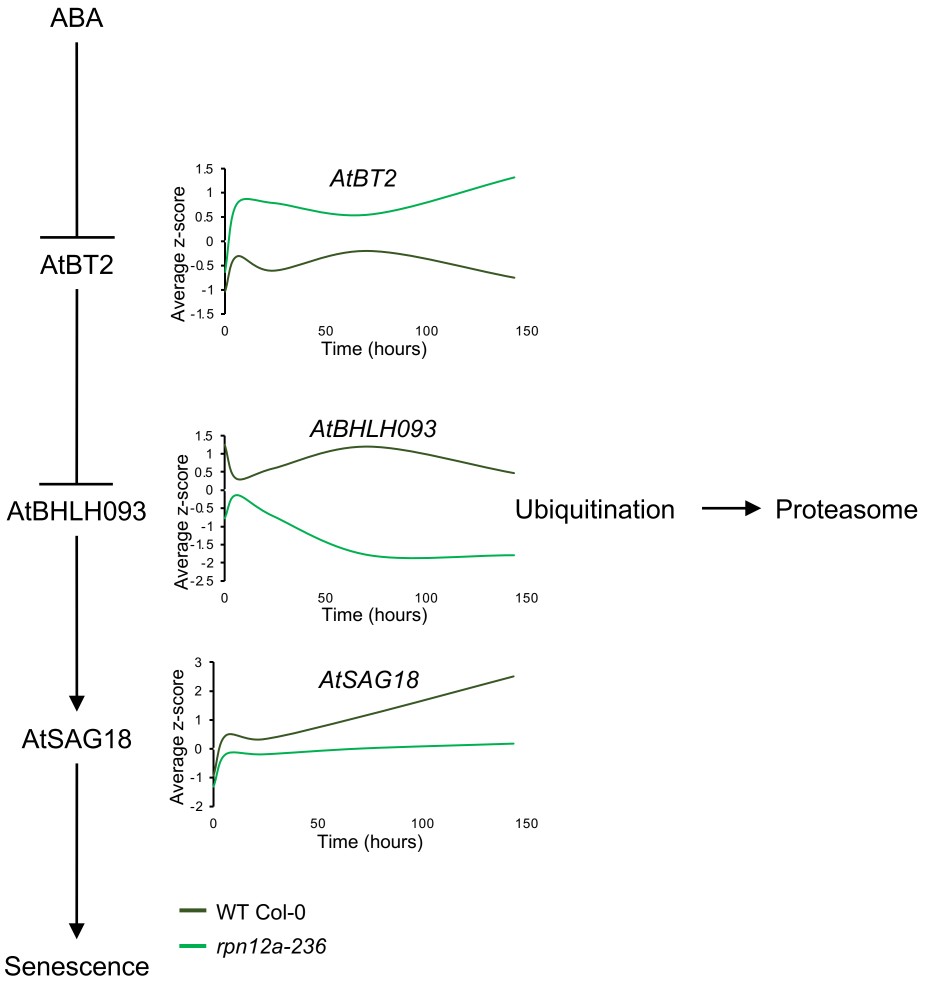

Figure S8. Current model for the regulation of senescence through the action of AtBT2. In this model, AtBHLH093 activates the transcription of *AtSAG18*. AtBT2 induces the ubiquitination and subsequent degradation of AtBHLH093 by the proteasome delaying leaf senescence (model adapted from An et al. (2018) in *Malus domestica*).

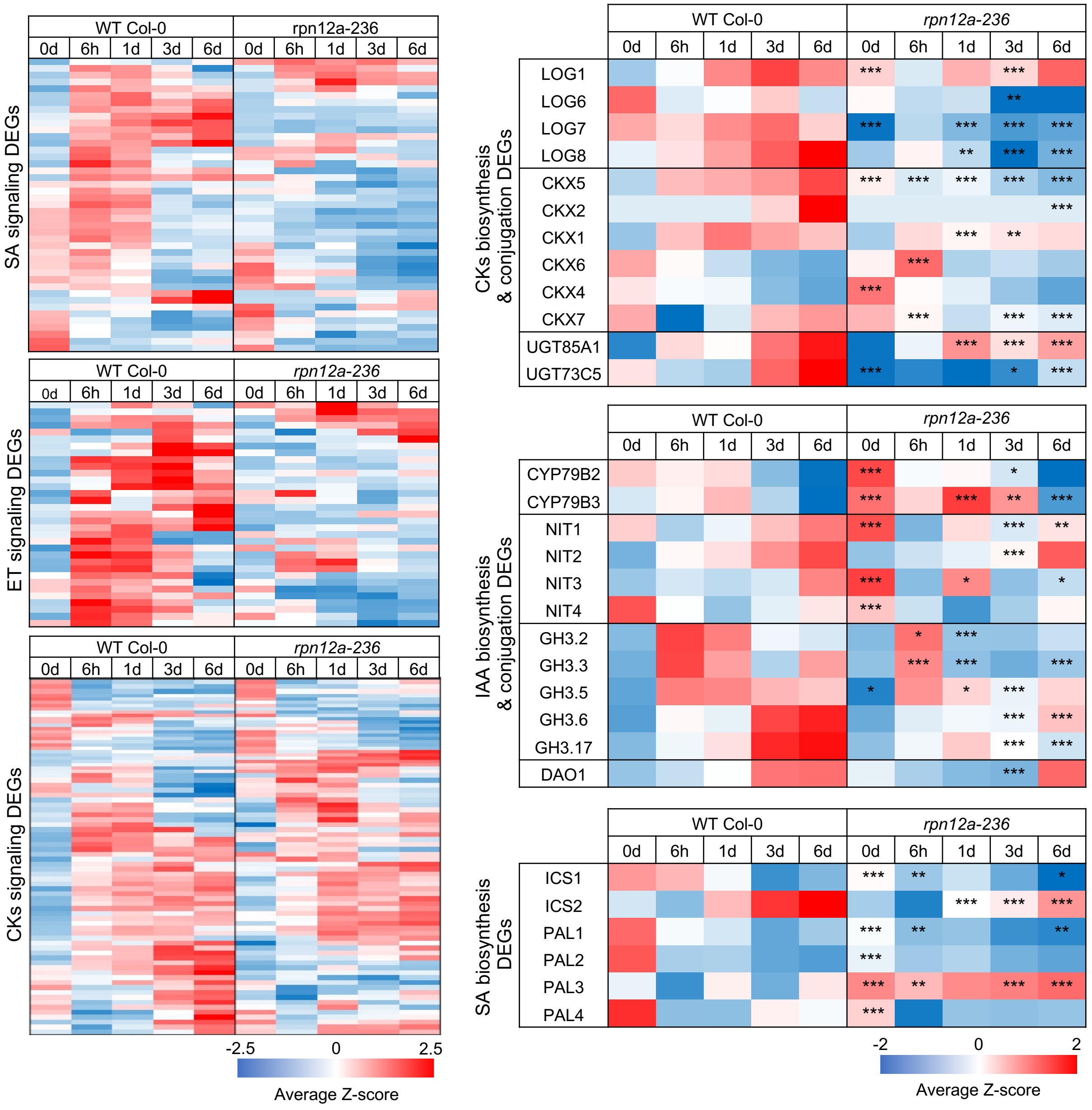

Figure S9. Transcriptomic analysis in WT Col-0 and *rpn12a-236* of genes associated with biosynthesis, conjugations and signalling of several hormones involved during leaf senescence. Heatmaps of SA, ET, IAA and CKs DEGs for at least one time point. On the left: signaling DEGs. On the right: biosynthesis and conjugation DEGs. A Student's t-test showed statistically significant differences at * p< 0.01, ** p<0.001, *** p<0.0001

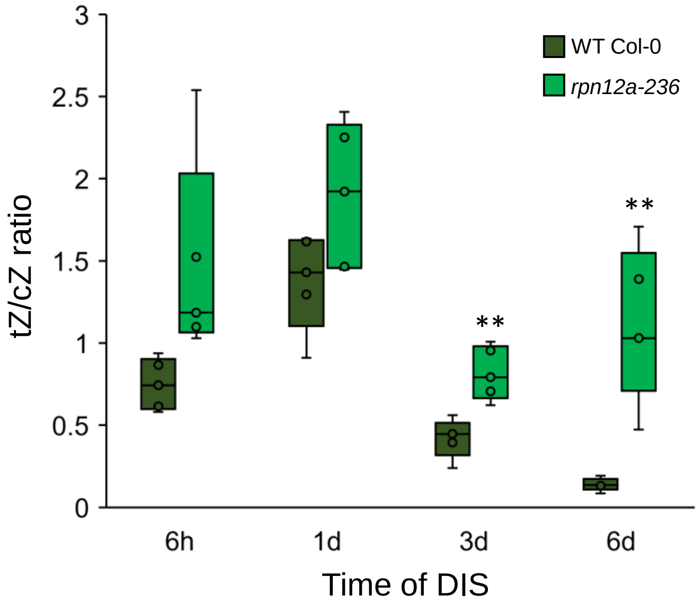

Figure S10. tZ/cZ ratio. Values obtained from the LC/MS were used to visualize the variation in the tZ/cZ ratio during DIS. The interquartile range of the box is from the 25th to 75th percentiles, and the middle line is the median; whiskers represent min and max values. A Student's t-test showed statistically significant differences at ** p<0.01.

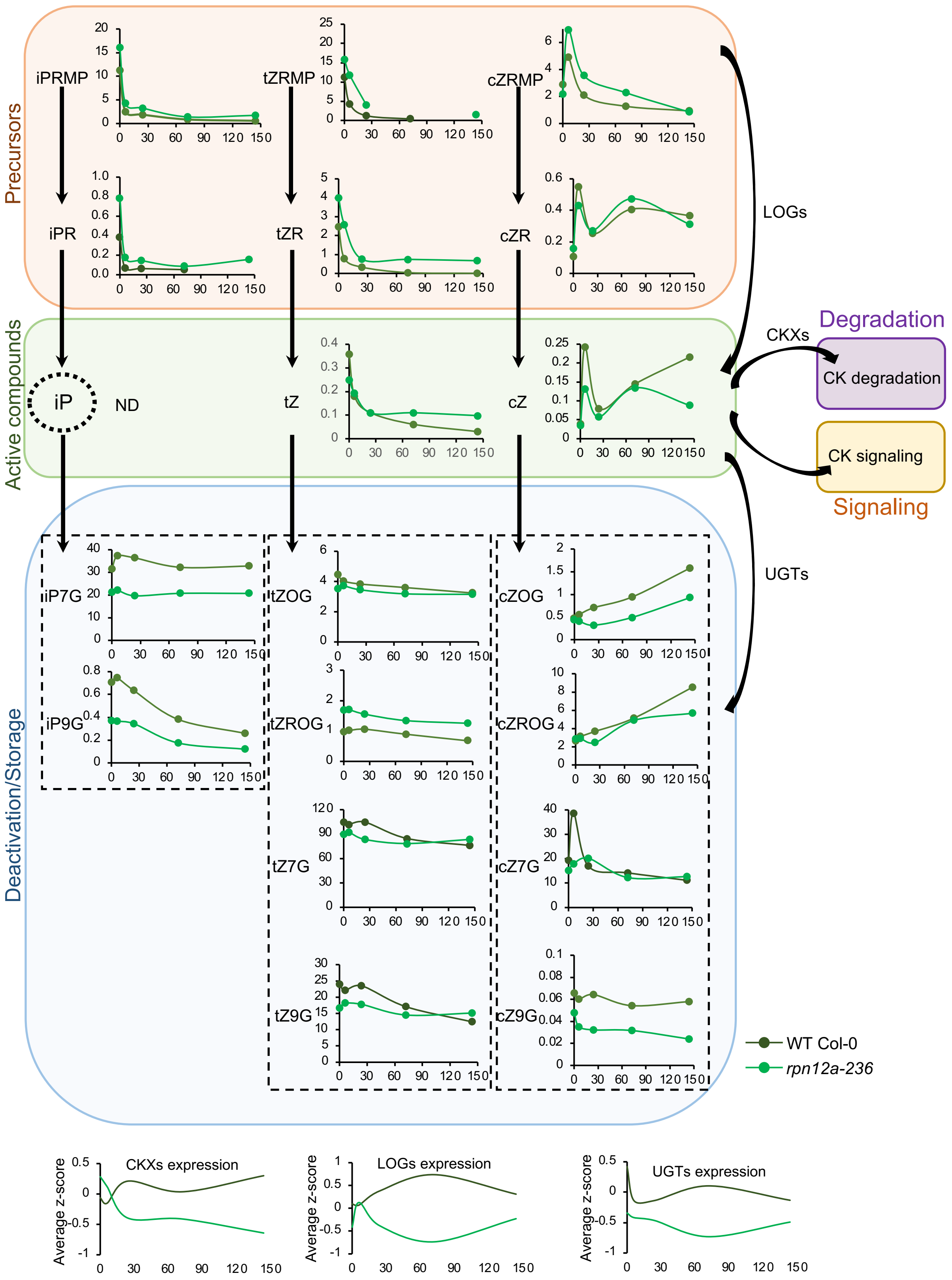

Figure S11. Cytokinin-related metabolites in response to DIS. . For each metabolite, total pools (in pmol/g FW) were plotted over time. Normalized average abundance of transcripts obtained from the RNAseq dataset are shown on the bottom heatmap. ND=not detected.

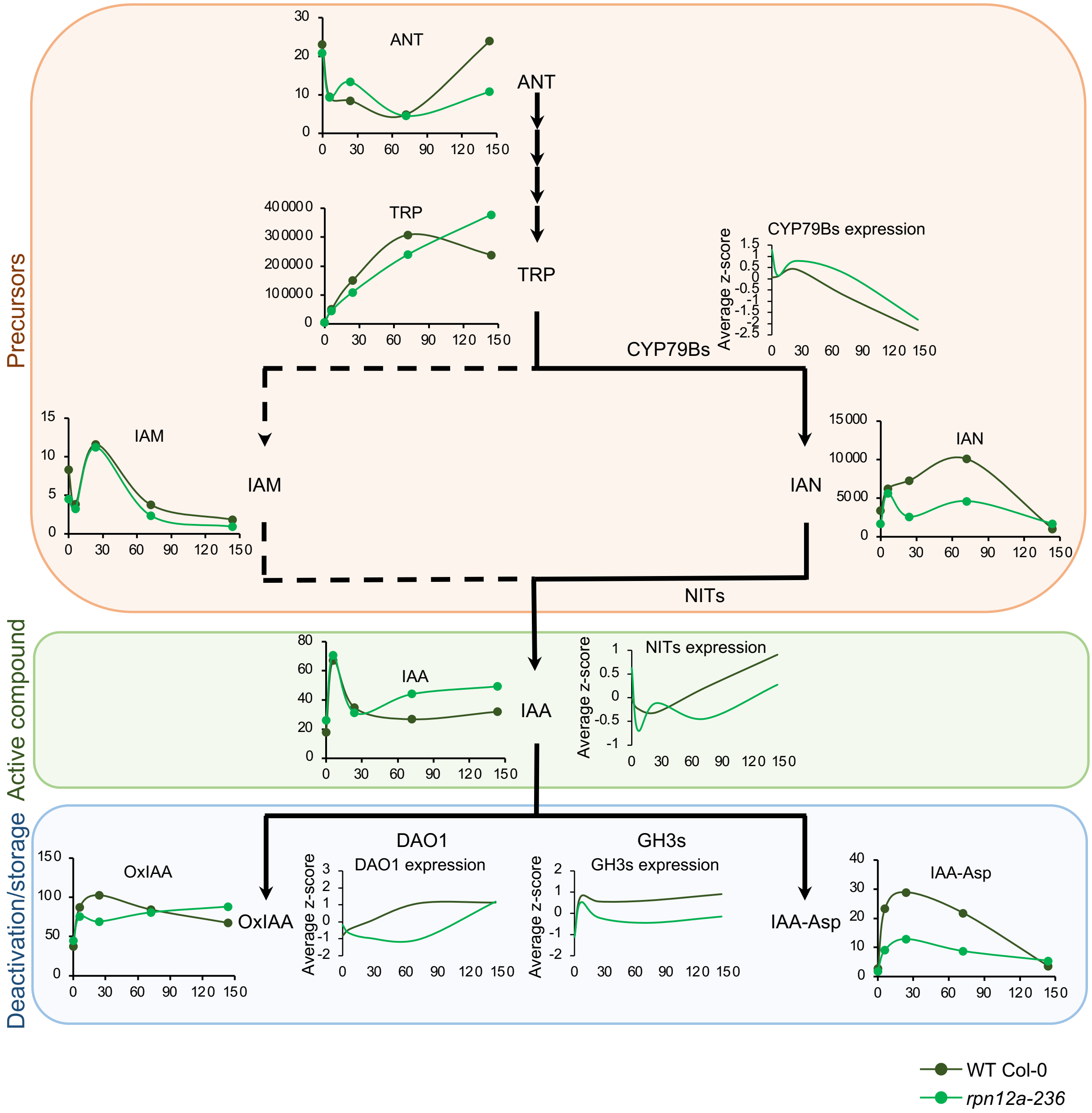

Figure S12. Auxin-related metabolites in response to DIS. For each metabolite, total pools (in pmol/g FW) were plotted over time. Normalized average abundance of transcripts obtained from the RNAseq dataset is shown on the bottom heatmap. Dashed lines indicate unclear metabolic paths.
