# Supplementary material for: The RPN12a proteasome subunit is essential for the multiple hormonal homeostasis controlling the progression of leaf senescence
Source: Commun Biol. 2022 Sep 30;5:1043. doi: 10.1038/s42003-022-03998-2 (PMC9525688; doi:10.1038/s42003-022-03998-2)
Supplement: Supplementary file 2 — Description of Additional Supplementary Files [file 42003_2022_3998_MOESM2_ESM.pdf]

Description of Additional Supplementary Files

File name: Supplementary Data 1

Description: List of homozygous mutations obtained from whole genome sequencing**.**

File name: Supplementary Data 2

Description: Proteasome subunits expression profile. The source data behind figure S7.

File name: Supplementary Data 3

Description: Senescence-responsive genes expression profile. The source data behind figure 5A**.**

File name: Supplementary Data 4

Description: Expression profile of genes involved in hormone biosynthesis, homeostasis and signalling. The source data behind figure S9.

File name: Supplementary Data 5

Description: Quantification of metabolites related to CKs, auxin, JA, SA, and ABA. The source data behind figures 6, S10, S11 and S12.

File name: Supplementary Data 6

Description: List of primers used.

File name: Supplementary Data 7

Description: All source data for graphs and charts.

File name: Supplementary Data 8

Description: Overview of RNAseq data, including raw and post-QC read counts and pseudo-alignment rates. The source data behind figures 4, 5, S6, S7, S8, S9.
