# Supplementary material for: The RPN12a proteasome subunit is essential for the multiple hormonal homeostasis controlling the progression of leaf senescence
Source: Commun Biol. 2022 Sep 30;5:1043. doi: 10.1038/s42003-022-03998-2 (PMC9525688; doi:10.1038/s42003-022-03998-2)
Supplement: Supplementary file 10 — Supplementary Data 8 [file 42003_2022_3998_MOESM10_ESM.pdf]

Arabidopsis fsg project - biological QA

Nicolas Delhomme & Clement Boussardon

2021-11-29

- 1 Setup
  - 1.1 Loading data
  - 1.2 Create the DESeq object
  - 1.3 Variance Stabilising Transformation
- 2 Quality Assessment
  - 2.1 Principal Component Analysis
  - 2.2 Two dimensions plots
  - 2.3 Expressed genes
    - 2.3.1 Hierarchical clustering of the data
    - 2.3.2 Heatmap
- 3 Session Info

1 Setup

1.1 Loading data

Set the working dir

```
setwd("/mnt/picea/projects/arabidopsis/okeech/arabidopsis-fsg")
```

Librarius

```
suppressPackageStartupMessages(library(DESeq2)) # Normalisation
suppressPackageStartupMessages(library(vsn))
suppressPackageStartupMessages(library(gplots))
suppressPackageStartupMessages(library(RColorBrewer)) # Large color range
suppressPackageStartupMessages(library(tximport)) # Read Kallisto files
suppressPackageStartupMessages(library(scatterplot3d))
```

Define a palette (8 colors)

```
pal <- brewer.pal(8,"Dark2")
```

Save the default margin parameters

```
mar=par("mar")
```

source a few helper scripts

```r
source("~/Git/UPSCb/src/R/featureSelection.R")
source("~/Git/UPSCb/src/R/plot.multidensity.R")
```

Read the sample file tab delimited

```r
samples <- read.delim("~/Git/UPSCb/projects/arabidopsis-fsg/doc/531vsRpn10vsCol_RNAse
q.txt")
samples531 <- samples [-c(28:42),]
```

Load dataset

```r
countsRpn12a <- read.csv ("analysis/kallisto/raw-unormalised-gene-expression_data.cs
v", header=T, row.names=1)
length(rowSums(countsRpn12a)) #32833 genes
```

```
## [1] 32833
```

```r
length(which(rowSums(countsRpn12a) == 0)) #5783 genes without counts
```

```
## [1] 5783
```

```r
geneswithcount <- which(rowSums(countsRpn12a) != 0) #genes with expression
write.csv(geneswithcount,file="analysis/kallisto/genes_with_counts531.csv")
```

The cumulative transcript coverage is deep, about 1000X

```r
countsRpn12a <- as.matrix (countsRpn12a)
plot(density(log10(rowMeans(countsRpn12a))),col=pal[1],
     main="gene mean raw counts distribution",
     xlab="mean raw counts (log10)")
```

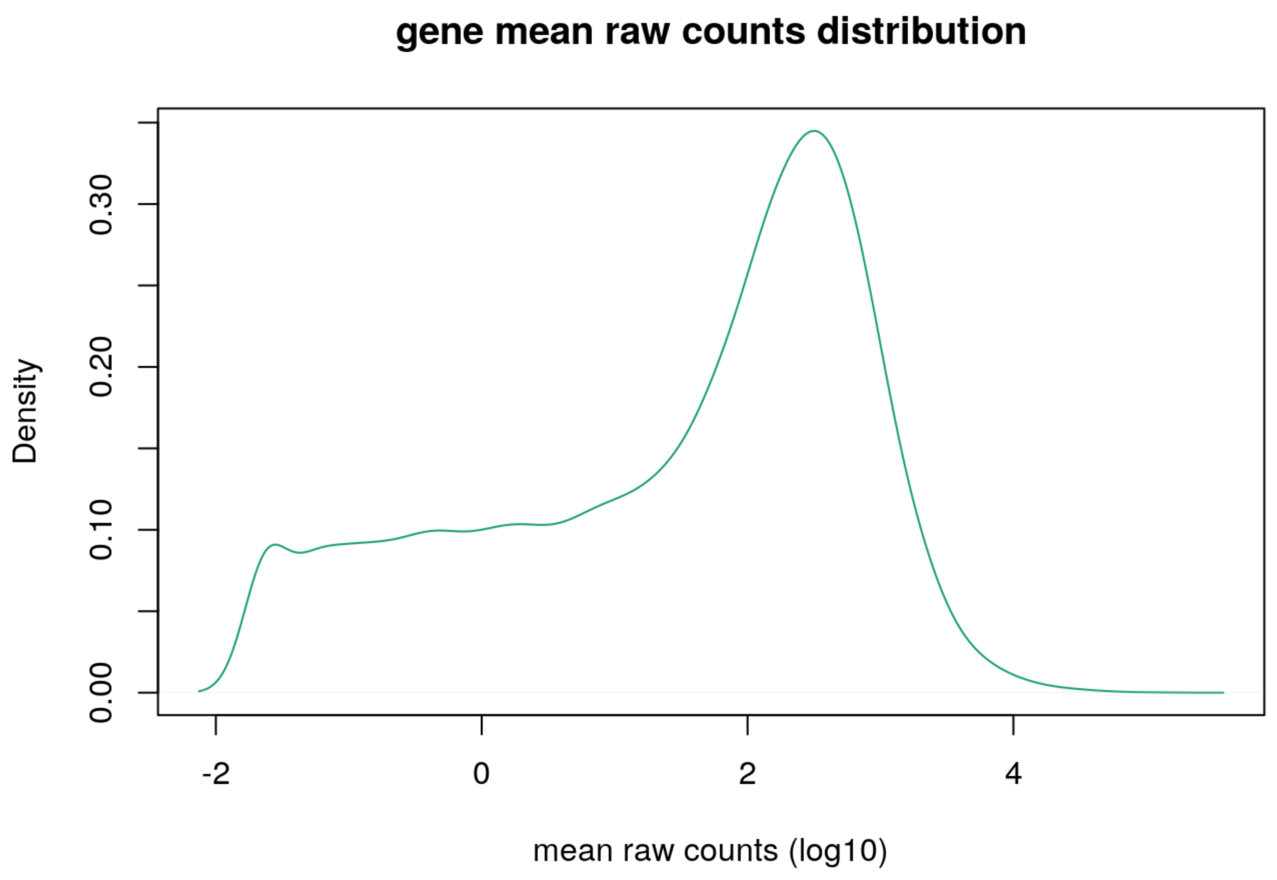


```
samples531 <- within(samples531, {
  Line <- as.factor(Line)
  Treatment <- as.factor(Treatment)
  Hours <- as.factor(Hours)
})
```

The same is done for the individual samples colored by treatment. The samples are extremely similar. Light and IDL show a similar distribution for all samples.

```
plot.multidensity(lapply(1:ncol(countsRpn12a),function(k){log10(countsRpn12a)[,k]}),
                  col=pal[as.integer(samples531$Treatment)],
                  legend.x="topright",
                  legend=levels(samples531$Treatment),
                  legend.col=pal[1:nlevels(samples531$Treatment)],
                  legend.lwd=2,
                  main="sample raw counts distribution",
                  xlab="per gene raw counts (log10)")
```

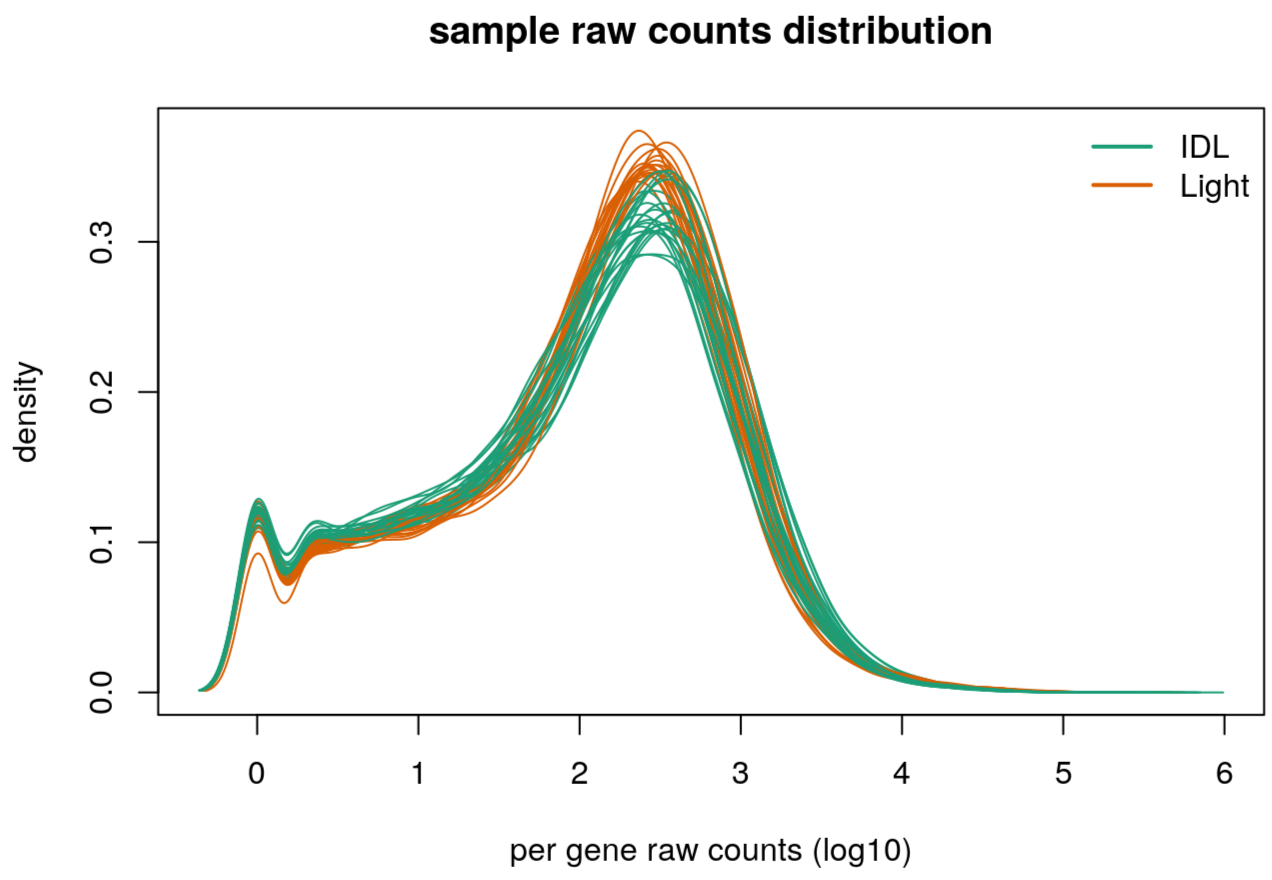

And colored by line

```
# Genotypes show a similar distribution for all samples.
plot.multidensity(lapply(1:ncol(countsRpn12a),function(k){log10(countsRpn12a)[,k]}),
                  col=pal[as.integer(samples531$Line)],
                  legend.x="topright",
                  legend=levels(samples531$Line),
                  legend.col=pal[1:nlevels(samples531$Line)],
                  legend.lwd=2,
                  main="sample raw counts distribution",
                  xlab="per gene raw counts (log10)")
```

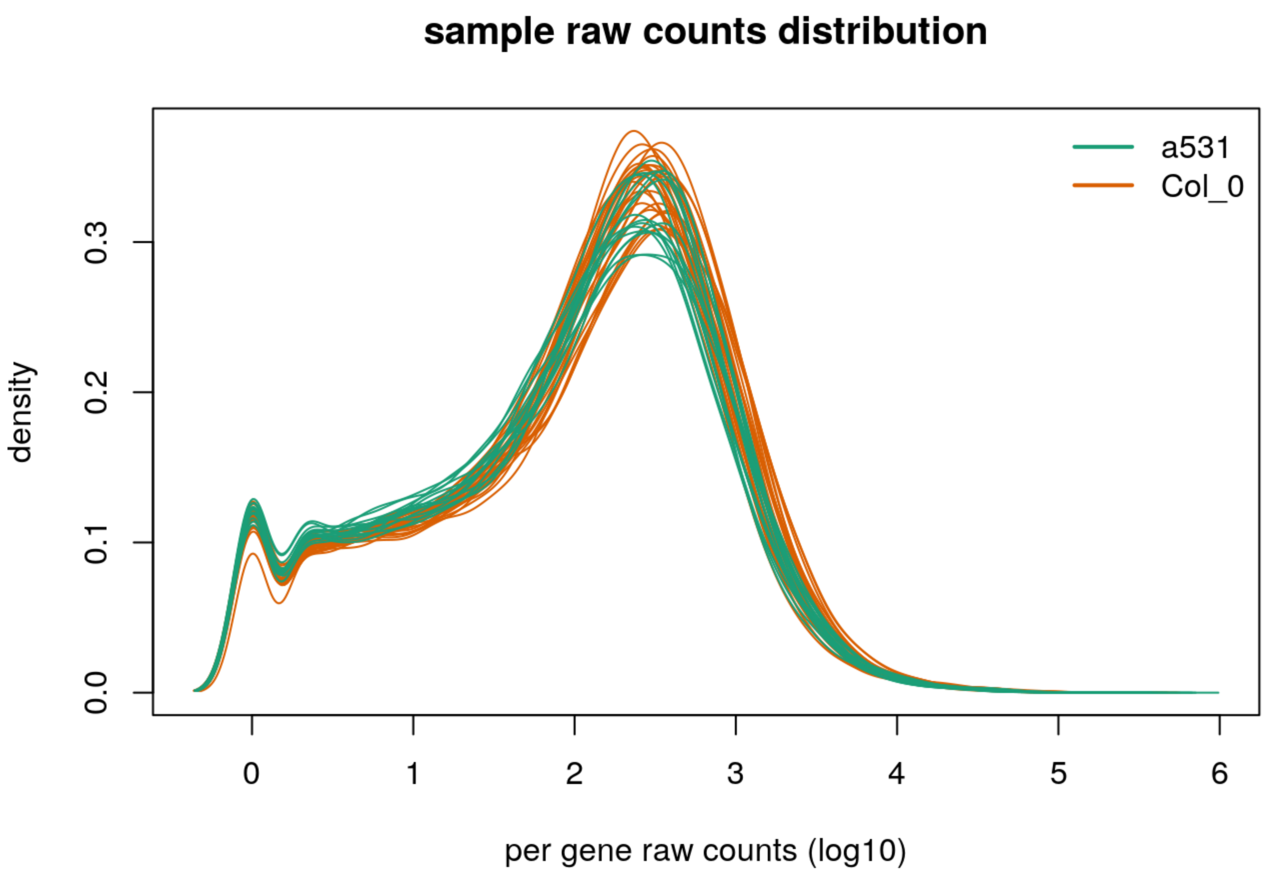

1.2 Create the DESeq object

First, reorder the Line and Treatment factors

```
samples531$Line <- relevel(samples531$Line,"Col_0")
samples531$Treatment <- relevel(samples531$Treatment,"Light")

dds531 <- DESeqDataSetFromMatrix(countsRpn12a,
                                 samples531,
                                 ~Line*Hours)
```

Attach sample information

```
save(dds531,file="analysis/kallisto/DESeq-object-LineByHours_rpn12a.rda")
```

Estimating the size factor There is little difference in the size factor (0.8-1.3).

```
dds531 <- estimateSizeFactors(dds531)
boxplot(colData(dds531)$sizeFactor,main="Library size factor",ylab="proportion")
abline(h=1,lty=2)
```

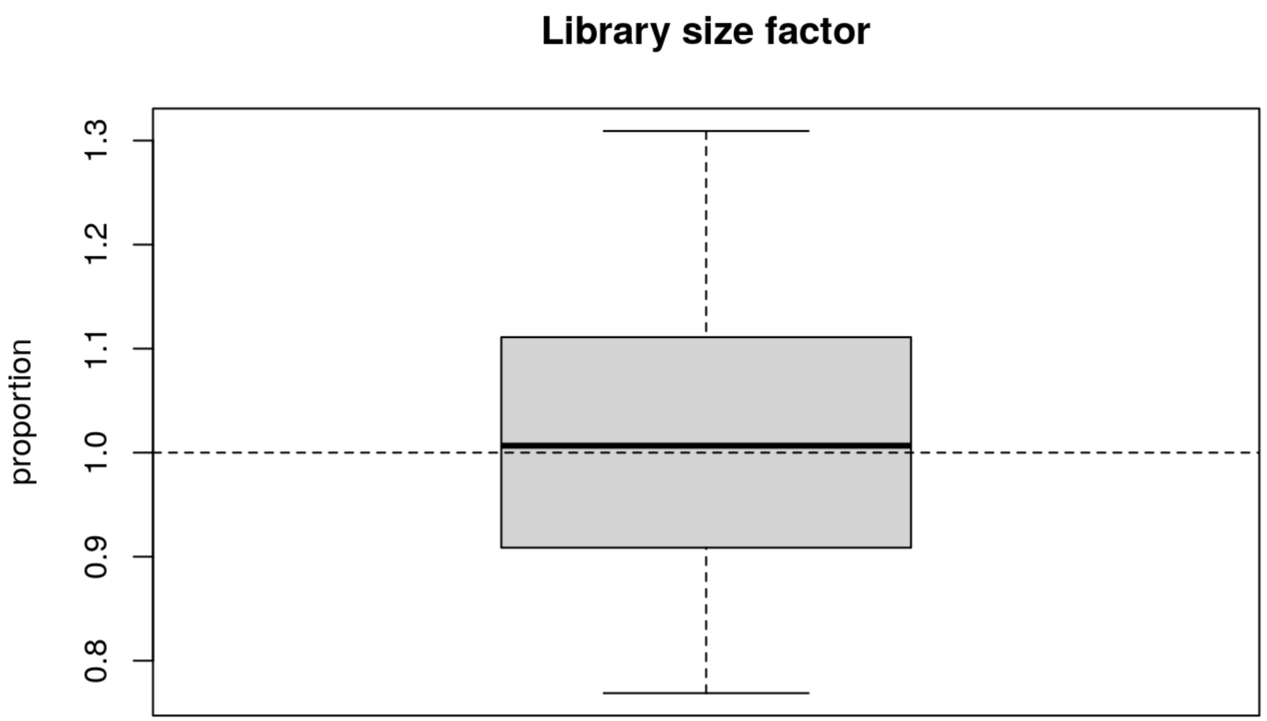

1.3 Variance Stabilising Transformation

Since there is almost no difference in library size, a VST is perfectly applicable. This is the situation prior to normalisation

```
meanSdPlot(log2(counts(dds531)[rowSums(counts(dds531)) > 0,]))
```

```
## Warning: Removed 10217 rows containing non-finite values (stat_binhex).
```

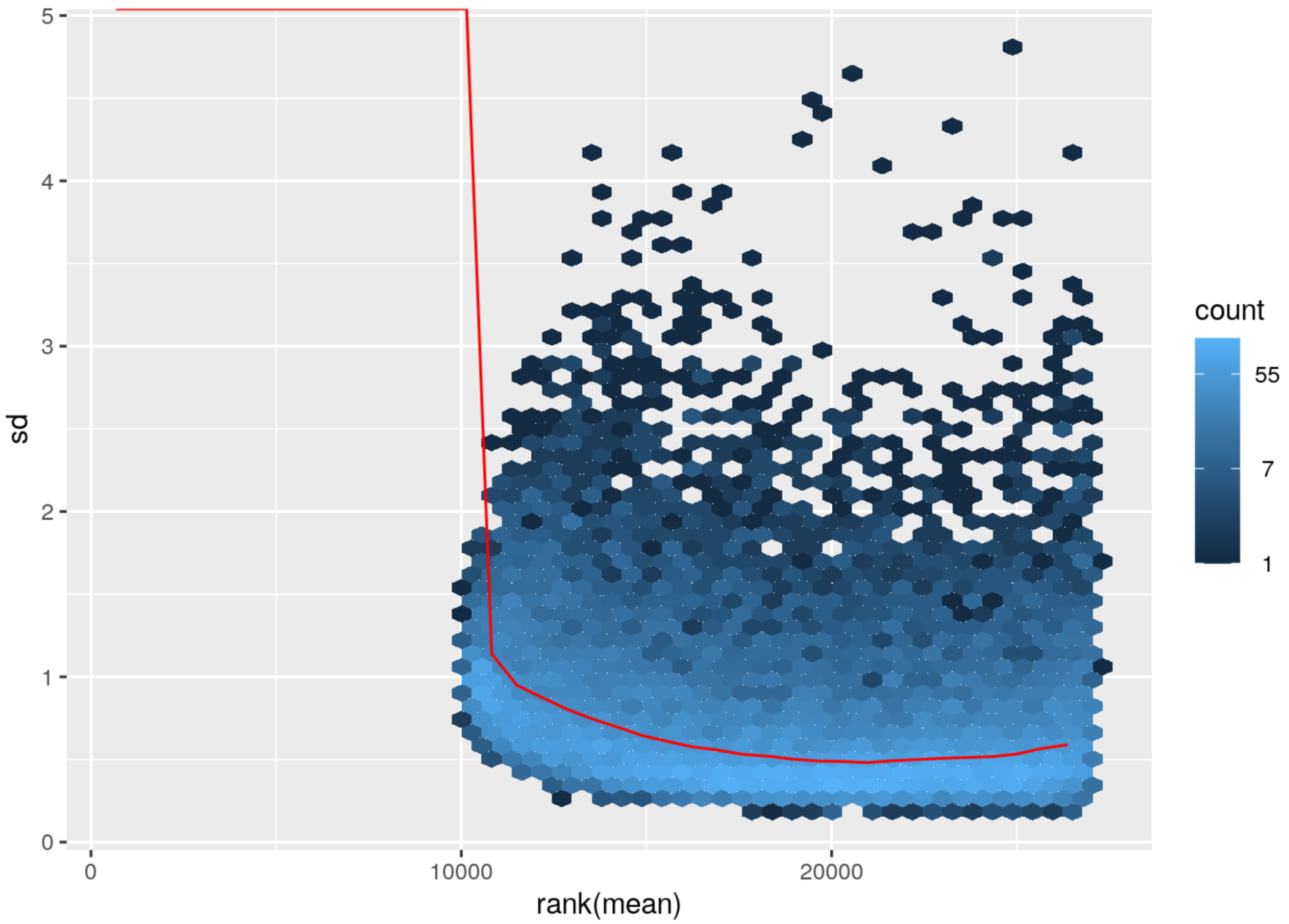

Normalisation - blind, we give no prior as we want to assess quality (heteroscedastic)

```
vst531 <- varianceStabilizingTransformation(dds531,blind=TRUE)
```

Extract the normalised counts

```
vsd531 <- assay(vst531)
```

Look at the VST fit. It looks ok, around 0.5 sd on average (variation 0.1 to 0.7). Visualize the corrected mean - sd relationship. It is fairly linear, meaning we can assume homoscedasticity. The slight initial trend / bump is due to genes having few counts in a few subset of the samples and hence having a higher variability. This is expected.

```
meanSdPlot(vsd531[rowSums(counts(dds531)) > 0,])
```

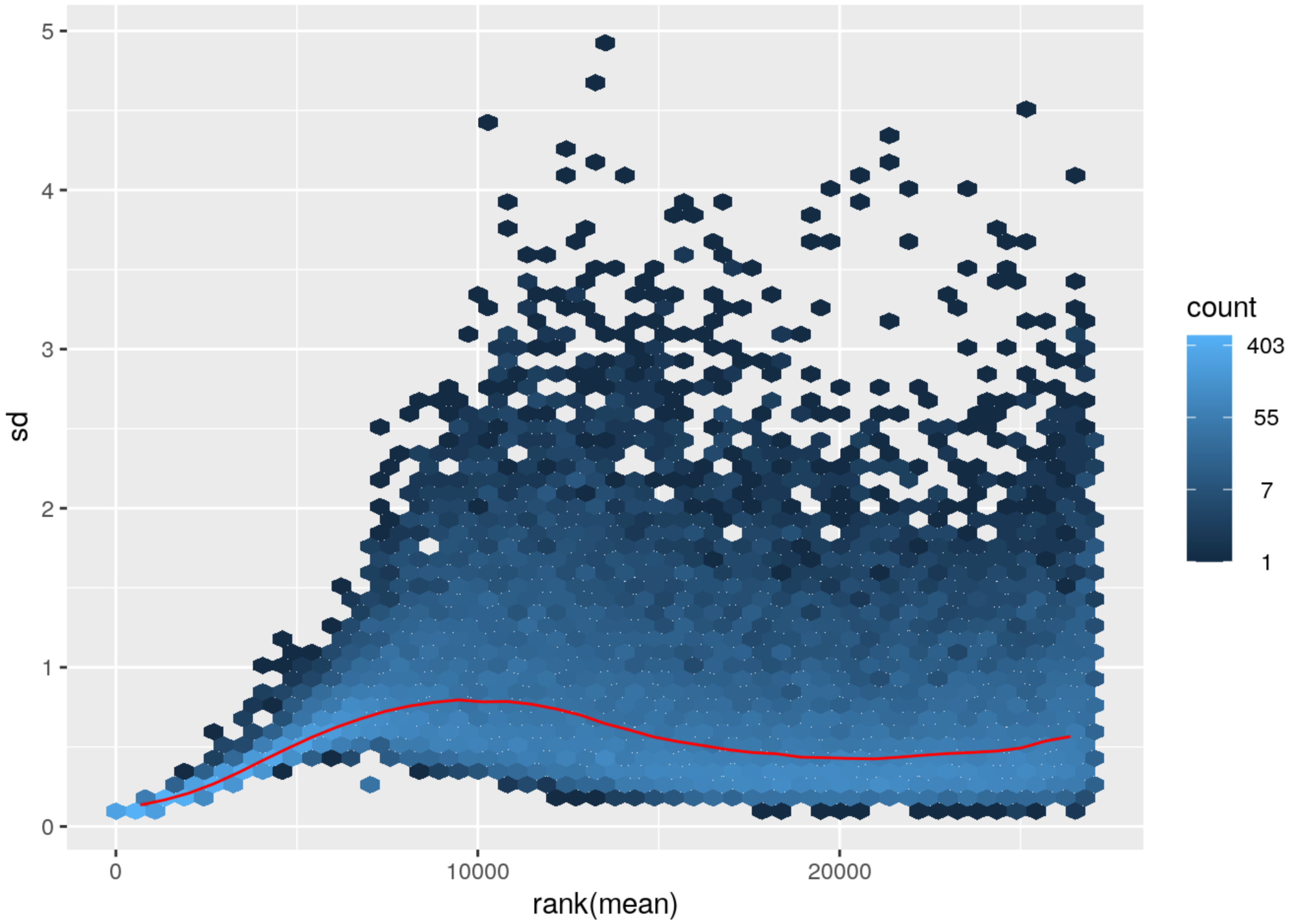

The VST introduces an offset

```
range(vsd531)
```

```
## [1]  1.692855 19.078862
```

Which we remove, so that 0 means no expression

```
vsd531 <- vsd531 - min(vsd531)
write.csv(vsd531, "analysis/kallisto/vst-blind-normalized-gene-expression_data-531.cs
v")
```

Transform to z-score

```
vstz531 <- t(scale(t(vsd531)))
write.csv(vstz531,"analysis/kallisto/z-score_transformation_531.csv")
```

2 Quality Assessment

2.1 Principal Component Analysis

```
# PC1=49%, PC2=13%, PC3=10%, PC4=5%
pc <- prcomp(t(vsd531))
percent <- round(summary(pc)$importance[2,]*100)
```

2.2 Two dimensions plots

Coloring Genotypes

```
plot(pc$x[,1],
     pc$x[,2],
     xlab=paste("Comp. 1 (",percent[1],"%)",sep=""),
     ylab=paste("Comp. 2 (",percent[2],"%)",sep=""),
     pch=c(19,17)[as.integer(samples531$Treatment)],
     col=pal[as.integer(samples531$Line)])
legend("topleft",pch=19,
       col=pal[1:nlevels(as.factor(samples531$Line))],
       legend=levels(as.factor(samples531$Line)))
legend("topright",pch=as.numeric(unique(samples531$Treatment)),
       legend=levels(factor(samples531$Treatment)))
```

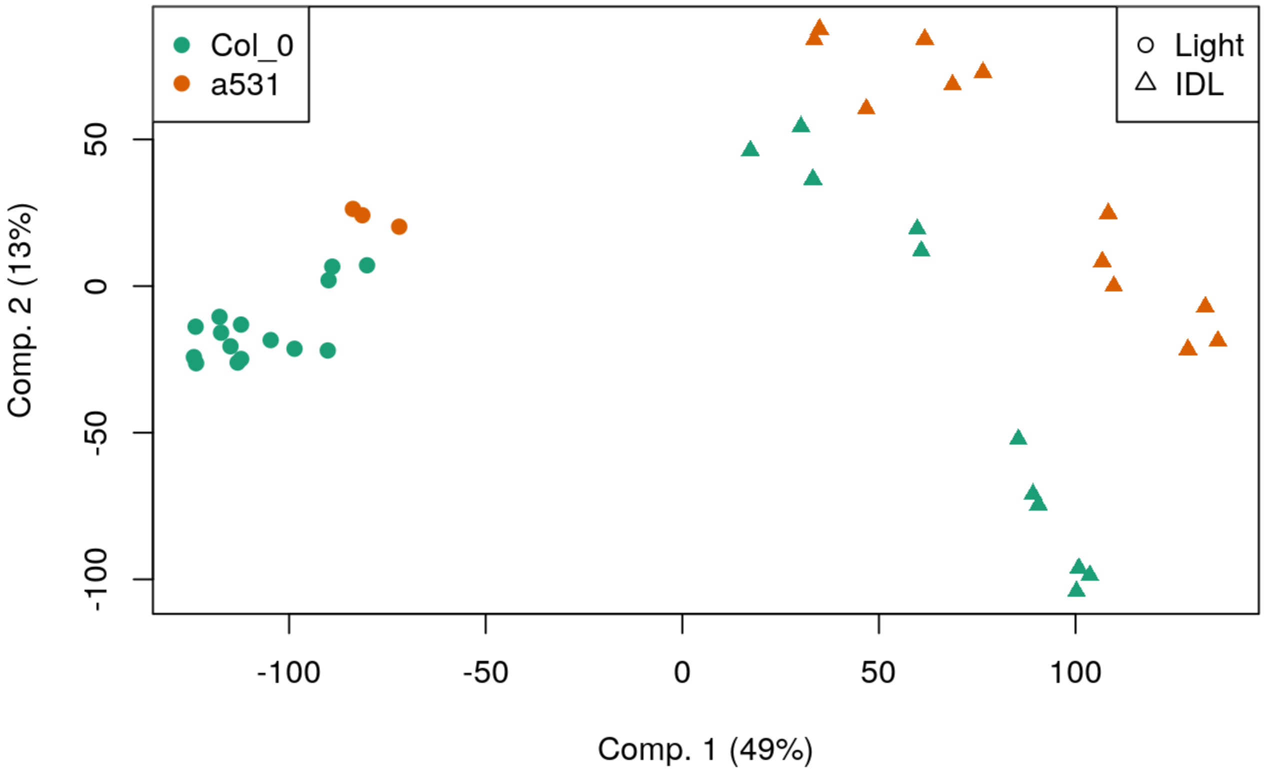

```
plot(pc$x[,1],
     pc$x[,3],
     xlab=paste("Comp. 1 (",percent[1],"%)",sep=""),
     ylab=paste("Comp. 3 (",percent[3],"%)",sep=""),
     pch=c(19,17)[as.integer(samples531$Treatment)],
     col=pal[as.integer(samples531$Line)])
legend("topleft",pch=19,
       col=pal[1:nlevels(as.factor(samples531$Line))],
       legend=levels(as.factor(samples531$Line)))
legend("topright",pch=as.numeric(unique(samples531$Treatment)),
       legend=levels(factor(samples531$Treatment)))
```

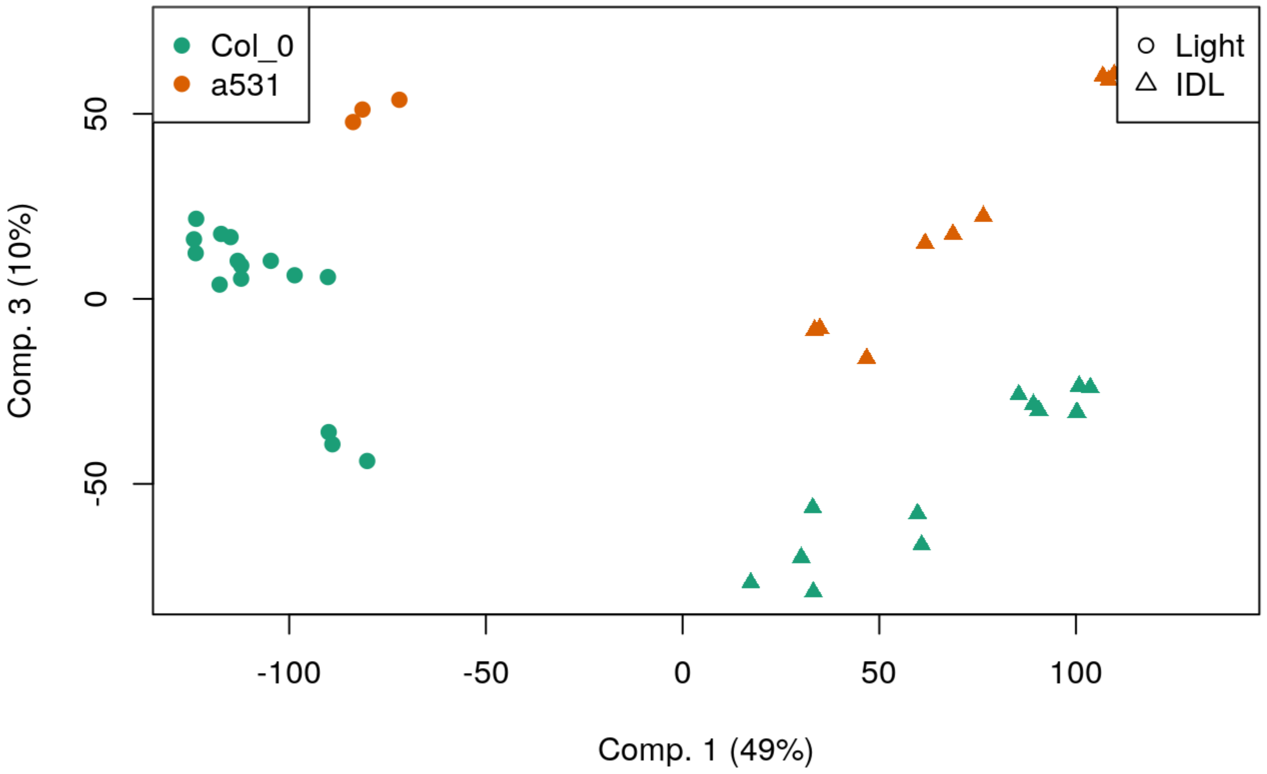


```
plot(pc$x[,2],
     pc$x[,3],
     xlab=paste("Comp. 2 (",percent[2],"%)",sep=""),
     ylab=paste("Comp. 3 (",percent[3],"%)",sep=""),
     pch=c(19,17)[as.integer(samples531$Treatment)],
     col=pal[as.integer(samples531$Line)])
legend("topleft",pch=19,
       col=pal[1:nlevels(as.factor(samples531$Line))],
       legend=levels(as.factor(samples531$Line)))
legend("topright",pch=as.numeric(unique(samples531$Treatment)),
       legend=levels(factor(samples531$Treatment)))
```

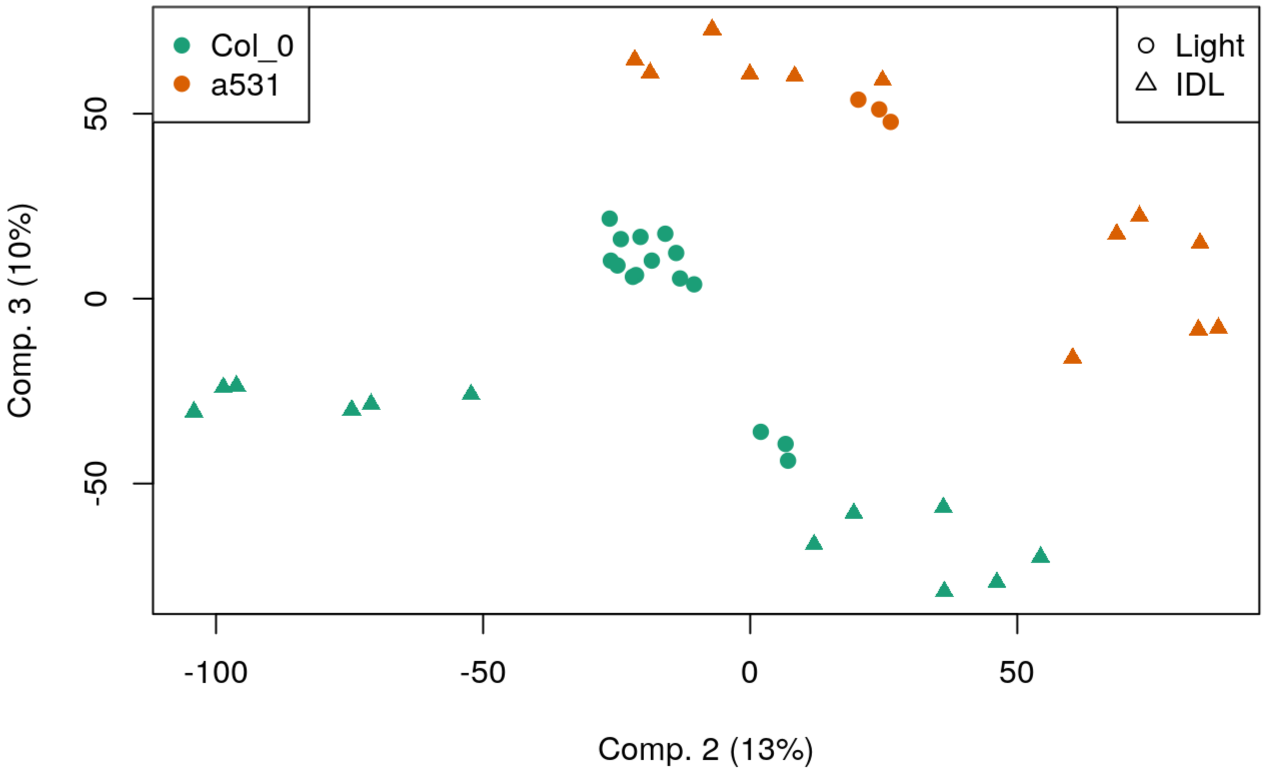

Coloring Hours (instead of Treatments)

```
f<-relevel (samples531$Hours,"6h")
samples531$Hours <- relevel(f,"0d")

plot(pc$x[,1],
     pc$x[,2],
     xlab=paste("Comp. 1 (",percent[1],"%)",sep=""),
     ylab=paste("Comp. 2 (",percent[2],"%)",sep=""),
     pch=c(19,3,17)[as.integer(samples531$Line)],
     col=pal[as.integer(samples531$Hours)])
legend("topleft",pch=19,
       col=pal[1:nlevels(as.factor(samples531$Hours))],
       legend=levels(as.factor(samples531$Hours)))
legend("topright",pch=as.numeric(unique(samples531$Line)),
       legend=levels(factor(samples531$Line)))
text(pc$x[,1],
     pc$x[,2],
     labels=samples$Rep,cex=.5,adj=-.3)
```

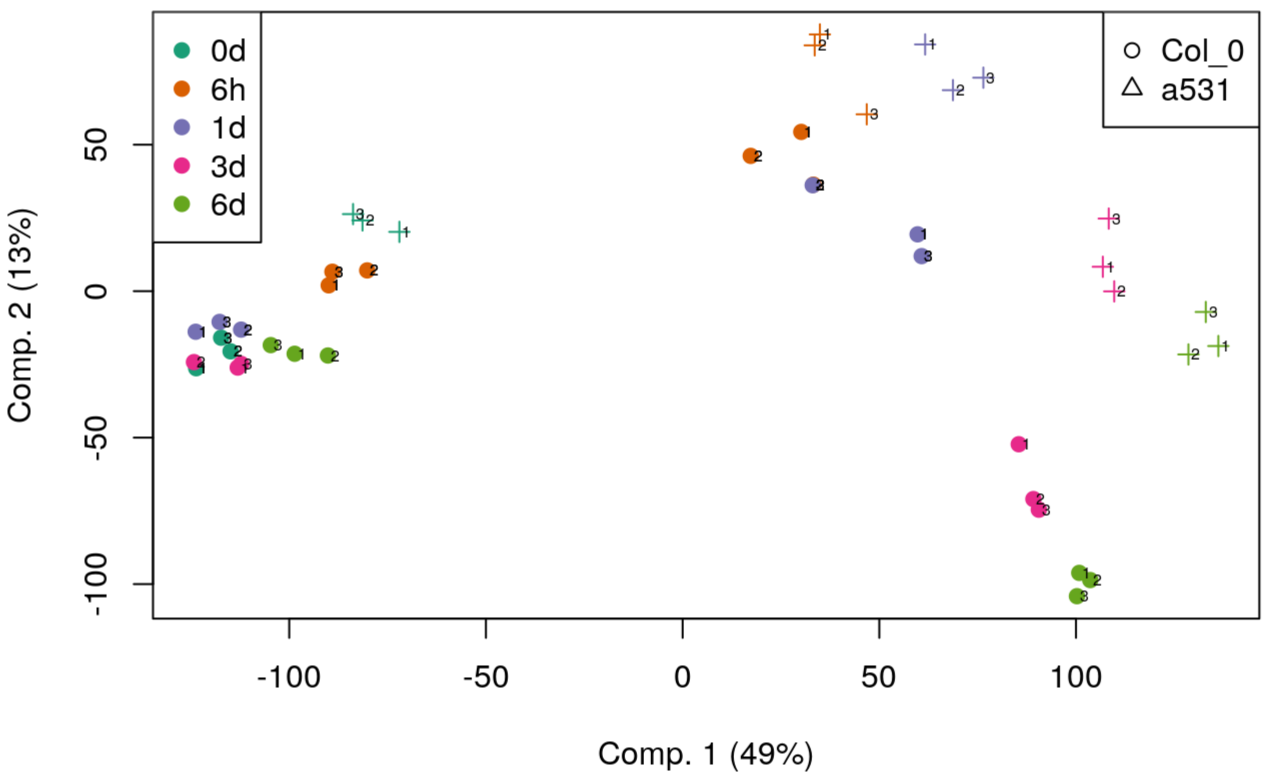

2.3 Expressed genes

```
sels <- sapply(1:10,function(i){
  featureSelect(vsd531,conditions = factor(paste0(samples531$Line,
                                                   samples531$Treatment,
                                                   samples531$Hours)),
                exp=i)})
```

a cutoff at 2 seems reasonable

```
plot(colSums(sels),type="l",xlab="vst cutoff",
     main="number of genes selected at cutoff",ylab="number of genes")
```

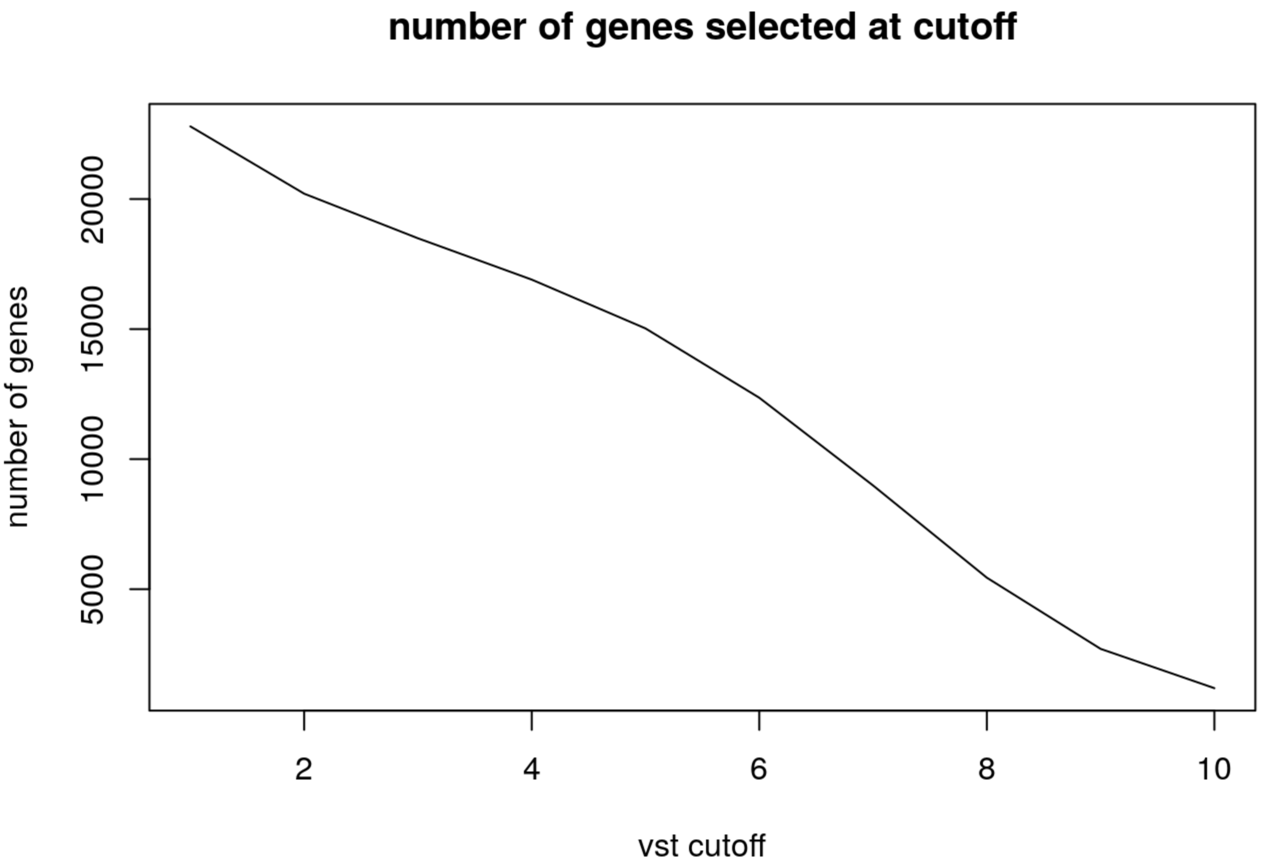


```
sel <- sels[,2]
```

2.3.1 Hierarchical clustering of the data

```
plot(hclust(dist(t(vsd531[sel,]))),
     labels=paste(samples531$Line,samples531$Treatment,
                  samples531$Hours,sep="_"))
```

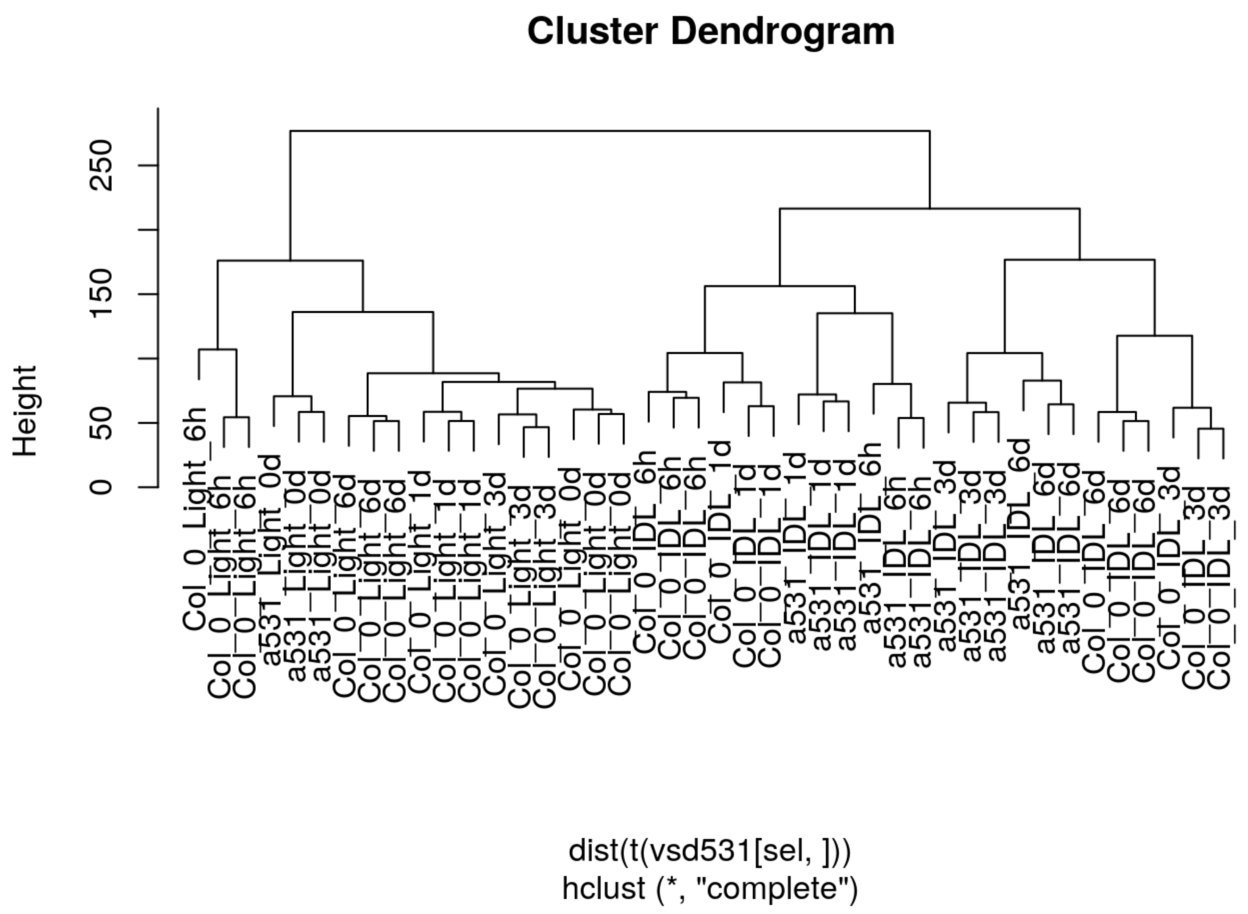

2.3.2 Heatmap

Create a heatmap

```
hpal <- colorRampPalette(c("blue","white","red"))(100)
```

z scale for gene counts (difference between mean expression and sample expression)

```
s.vst <- t(scale(t(vsd531)))

library(hyperSpec)
```

```
## Loading required package: lattice
```

```
## Loading required package: grid
```

```
## Loading required package: ggplot2
```

```
## Loading required package: xml2
```

```
## Package hyperSpec, version 0.100.0
##
## To get started, try
##    vignette ("hyperspec")
##    package?hyperSpec
##    vignette (package = "hyperSpec")
##
## If you use this package please cite it appropriately.
##    citation("hyperSpec")
## will give you the correct reference.
##
## The project homepage is http://hyperspec.r-forge.r-project.org
```

```
##
## Attaching package: 'hyperSpec'
```

```
## The following object is masked from 'package:IRanges':
##
##     collapse
```

```
heatmap.2(s.vst[sel,],distfun = pearson.dist,
          hclustfun = function(X){hclust(X,method="ward.D")},
          trace="none",col=hpal,labRow = FALSE,
          labCol=paste(samples531$Line,samples531$Treatment,
                       samples531$Hours,sep="_"),
          cexCol = 0.8,
          margins=c(7.1,0.1))
```

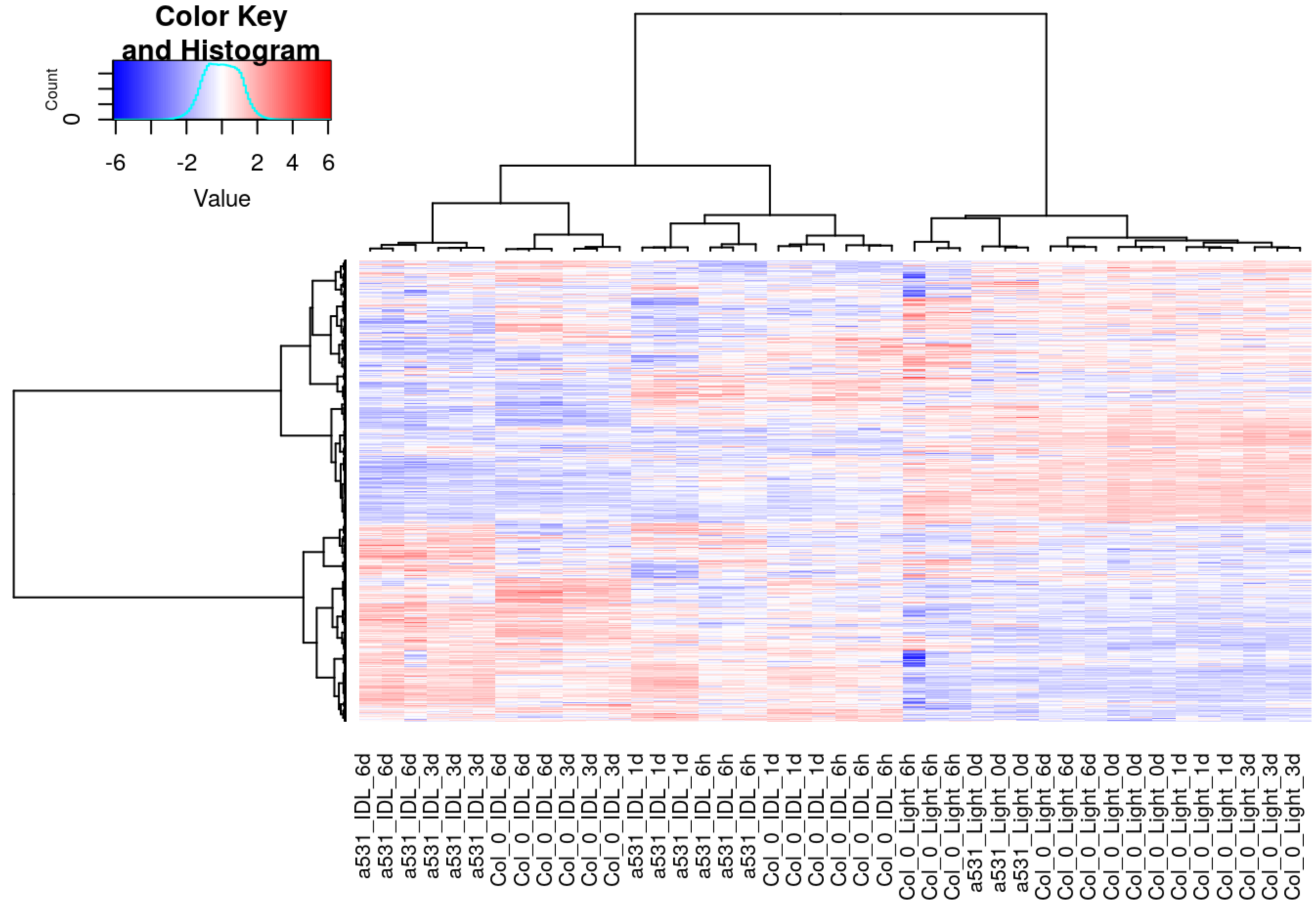

3 Session Info

```
## R version 4.1.1 (2021-08-10)
## Platform: x86_64-pc-linux-gnu (64-bit)
## Running under: Ubuntu 18.04.6 LTS
##
## Matrix products: default
## BLAS/LAPACK: /usr/lib/x86_64-linux-gnu/libopenblasp-r0.2.20.so
##
## locale:
##  [1] LC_CTYPE=en_US.UTF-8       LC_NUMERIC=C
##  [3] LC_TIME=en_US.UTF-8        LC_COLLATE=en_US.UTF-8
##  [5] LC_MONETARY=en_US.UTF-8    LC_MESSAGES=en_US.UTF-8
##  [7] LC_PAPER=en_US.UTF-8       LC_NAME=C
##  [9] LC_ADDRESS=C               LC_TELEPHONE=C
## [11] LC_MEASUREMENT=en_US.UTF-8 LC_IDENTIFICATION=C
##
## attached base packages:
## [1] grid      stats4    stats     graphics  grDevices utils     datasets
## [8] methods   base
##
## other attached packages:
##  [1] hyperSpec_0.100.0           xml2_1.3.2
##  [3] ggplot2_3.3.5               lattice_0.20-45
##  [5] scatterplot3d_0.3-41        tximport_1.22.0
##  [7] RColorBrewer_1.1-2          gplots_3.1.1
##  [9] vsn_3.62.0                  DESeq2_1.34.0
## [11] SummarizedExperiment_1.24.0 Biobase_2.54.0
## [13] MatrixGenerics_1.6.0        matrixStats_0.61.0
## [15] GenomicRanges_1.46.0        GenomeInfoDb_1.30.0
## [17] IRanges_2.28.0              S4Vectors_0.32.2
## [19] BiocGenerics_0.40.0
##
## loaded via a namespace (and not attached):
##  [1] bitops_1.0-7           bit64_4.0.5            httr_1.4.2
##  [4] tools_4.1.1            bslib_0.3.1            utf8_1.2.2
##  [7] R6_2.5.1               affyio_1.64.0          KernSmooth_2.23-20
## [10] lazyeval_0.2.2         DBI_1.1.1              colorspace_2.0-2
## [13] withr_2.4.2            tidyselect_1.1.1       bit_4.0.4
## [16] compiler_4.1.1         preprocessCore_1.56.0  DelayedArray_0.20.0
## [19] labeling_0.4.2         sass_0.4.0             caTools_1.18.2
## [22] scales_1.1.1           hexbin_1.28.2          genefilter_1.76.0
## [25] affy_1.72.0            stringr_1.4.0          digest_0.6.28
## [28] rmarkdown_2.11         XVector_0.34.0         jpeg_0.1-9
## [31] pkgconfig_2.0.3        htmltools_0.5.2        fastmap_1.1.0
## [34] limma_3.50.0           highr_0.9              rlang_0.4.12
## [37] RSQLite_2.2.8          farver_2.1.0           jquerylib_0.1.4
## [40] generics_0.1.1         jsonlite_1.7.2         BiocParallel_1.28.0
## [43] gtools_3.9.2           dplyr_1.0.7            RCurl_1.98-1.5
## [46] magrittr_2.0.1         GenomeInfoDbData_1.2.7 Matrix_1.3-4
## [49] Rcpp_1.0.7             munsell_0.5.0          fansi_0.5.0
## [52] lifecycle_1.0.1        stringi_1.7.5          yaml_2.2.1
## [55] zlibbioc_1.40.0        blob_1.2.2             parallel_4.1.1
```

```
## [58] crayon_1.4.2          Biostrings_2.62.0      splines_4.1.1
## [61] annotate_1.72.0       KEGGREST_1.34.0        locfit_1.5-9.4
## [64] knitr_1.36            pillar_1.6.4           geneplotter_1.72.0
## [67] XML_3.99-0.8          glue_1.5.0             evaluate_0.14
## [70] latticeExtra_0.6-29   BiocManager_1.30.16    png_0.1-7
## [73] vctrs_0.3.8           testthat_3.1.0         gtable_0.3.0
## [76] purrr_0.3.4           assertthat_0.2.1       cachem_1.0.6
## [79] xfun_0.28             xtable_1.8-4           survival_3.2-13
## [82] tibble_3.1.6          AnnotationDbi_1.56.1   memoise_2.0.0
## [85] ellipsis_0.3.2
```
