# Supplementary material for: The RPN12a proteasome subunit is essential for the multiple hormonal homeostasis controlling the progression of leaf senescence
Source: Commun Biol. 2022 Sep 30;5:1043. doi: 10.1038/s42003-022-03998-2 (PMC9525688; doi:10.1038/s42003-022-03998-2)
Supplement: Supplementary file 11 — Reporting Summary [file 42003_2022_3998_MOESM11_ESM.pdf]

nature portfolio

Corresponding author(s): Olivier Keech

Last updated by author(s): Sep 12, 2022

Reporting Summary

Nature Portfolio wishes to improve the reproducibility of the work that we publish. This form provides structure for consistency and transparency in reporting. For further information on Nature Portfolio policies, see our Editorial Policies and the Editorial Policy Checklist.

Statistics

For all statistical analyses, confirm that the following items are present in the figure legend, table legend, main text, or Methods section.

n/a	Confirmed	
☐	☒	The exact sample size (n) for each experimental group/condition, given as a discrete number and unit of measurement
☐	☒	A statement on whether measurements were taken from distinct samples or whether the same sample was measured repeatedly
☐	☒	The statistical test(s) used AND whether they are one- or two-sided<br>*Only common tests should be described solely by name; describe more complex techniques in the Methods section.*
☐	☒	A description of all covariates tested
☐	☒	A description of any assumptions or corrections, such as tests of normality and adjustment for multiple comparisons
☐	☒	A full description of the statistical parameters including central tendency (e.g. means) or other basic estimates (e.g. regression coefficient) AND variation (e.g. standard deviation) or associated estimates of uncertainty (e.g. confidence intervals)
☐	☒	For null hypothesis testing, the test statistic (e.g. F, t, r) with confidence intervals, effect sizes, degrees of freedom and P value noted<br>*Give P values as exact values whenever suitable.*
☒	☐	For Bayesian analysis, information on the choice of priors and Markov chain Monte Carlo settings
☐	☒	For hierarchical and complex designs, identification of the appropriate level for tests and full reporting of outcomes
☒	☐	Estimates of effect sizes (e.g. Cohen's d, Pearson's r), indicating how they were calculated

Our web collection on statistics for biologists contains articles on many of the points above.

Software and code

Policy information about availability of computer code

Data collection	For whole genome sequencing:<br>*CTAB extraction of gDNA<br>*DNA sequencing was performed at Novogene Technology Co., Ltd (Animal and Plant Resequencing (WGS)). Illumina HiSeq PE150<br><br>For RNA-seq:<br>*RNA was extracted with EZNA plant RNA kit following provider recommendations (Omega Bio-tek, R6834-01).<br>*RNA sequencing was performed at Novogene Technology Co., Ltd (HiSeq platforms, paired-end 150 bp sequencing strategy).
Data analysis	For RNA-seq analysis:<br>*FastQC v0.11.4.<br>*SortMeRNA v2.1<br>*Trimmomatic v0.36<br>*kallisto v0.43.0<br>*R v3.3.2<br>*Bioconductor v3.3 tximport package v.1.2.0<br>*Bioconductor DESeq2 package v1.14.1<br>*MeV (MultiExperiment Viewer) v4.9

For manuscripts utilizing custom algorithms or software that are central to the research but not yet described in published literature, software must be made available to editors and reviewers. We strongly encourage code deposition in a community repository (e.g. GitHub). See the Nature Portfolio guidelines for submitting code & software for further information.

Data

Policy information about availability of data

All manuscripts must include a data availability statement. This statement should provide the following information, where applicable:

- Accession codes, unique identifiers, or web links for publicly available datasets
- A description of any restrictions on data availability
- For clinical datasets or third party data, please ensure that the statement adheres to our policy

There is no restriction on data availability.
Source data are available in the supplementary material, or at specified repositories.

Field-specific reporting

Please select the one below that is the best fit for your research. If you are not sure, read the appropriate sections before making your selection.

☒ Life sciences ☐ Behavioural & social sciences ☐ Ecological, evolutionary & environmental sciences

For a reference copy of the document with all sections, see nature.com/documents/nr-reporting-summary-flat.pdf

Life sciences study design

All studies must disclose on these points even when the disclosure is negative.

Sample size	For most analyses of gene and protein expression and imaging, leaves from 7-week-old plants grown in SD were individually darkened for 0, 6h, 1d, 3d, 6d. IDL was performed to trigger a different senescence response between in WT (leaf turning yellow) and rpn12a-236 (stay-green). Arabidopsis thaliana Columbia-0 (Col-0) WT and mutant plants were grown under short-day photoperiod (SD: light 8h at 22°C, dark 16h at 17°C) at 65% relative humidity and 180 µmol m-2 s-1 photosynthetically active radiation (PAR) or long-day photoperiod (LD: light 16h at 22°C, dark 8h at 17°C) at 65% relative humidity and 150 µmol m-2 s-1 PAR on a mixture soil:vermiculite 3:1. For SD grown plants, at 5-7 weeks after sowing, two leaves of each plant were covered individually for IDL. For detached rosette leaves experiment, leaves from 3 independent randomized plants were incubated in a 3 mM MES solution for 7 days in darkness at room temperature. For developmental senescence, rosettes from 3 randomized replicates were collected every 7 days after the first flower bud is visible, corresponding to the 5.10 Arabidopsis growth.
Data exclusions	No data were excluded from the analysis.
Replication	All data were repeated at least three time to confirm reproducibility.
Randomization	Arabidopsis plants grown under the same conditions were randomly selected based on the size and healthiness, harvested and processed for RNA extraction and hormonomics.
Blinding	For the genetic analysis of the mutants, phenotyping and genotyping were separately processed. The phenotype information was first collected and matched with the genotype to confirm the phenotype-genotype correlation.

Reporting for specific materials, systems and methods

We require information from authors about some types of materials, experimental systems and methods used in many studies. Here, indicate whether each material, system or method listed is relevant to your study. If you are not sure if a list item applies to your research, read the appropriate section before selecting a response.

Materials & experimental systems

n/a	Involved in the study	
☐	☒	Antibodies
☒	☐	Eukaryotic cell lines
☒	☐	Palaeontology and archaeology
☒	☐	Animals and other organisms
☒	☐	Human research participants
☒	☐	Clinical data
☒	☐	Dual use research of concern

Methods

n/a	Involved in the study	
☒	☐	ChIP-seq
☒	☐	Flow cytometry
☒	☐	MRI-based neuroimaging

Antibodies

Antibodies used	anti-Ubiquitin antibody (1/1000 in TBS-T, Sigma-Aldrich, U5379)<br>anti-PBA1 antibody (1/3000 in TBS-T, Agrisera, AS19 4260)

anti-RPN6 antibody (1/2000 in TBS-T, Agrisera, AS15 2832A)
anti-RPN12a antibody (1/1000, Abcam, ab98959)

Validation

anti-RPN12a: https://www.abcam.com/rpn12a-antibody-ab98959.html
anti-PBA1: https://www.agrisera.com/en/artiklar/pba1.html
anti-RPN6: https://www.agrisera.com/en/artiklar/rpn6-26s-proteasome-non-atpase-regulatory-subunit-9-.html
anti-Ubiquitin: https://www.sigmaaldrich.com/SE/en/product/sigma/u5379
Antibodies are all commercially available and the information for validation can be found at the link.
For anti Ubiquitin, a smear corresponding to the polyubiquitinated proteins is observed as proteins of variable sizes can be ubiquitinated.
